# Distinctive structural properties of THB11, a pentacoordinate *Chlamydomonas reinhardtii* truncated hemoglobin with N- and C-terminal extensions

**DOI:** 10.1007/s00775-020-01759-2

**Published:** 2020-02-11

**Authors:** Dennis Huwald, Sabrina Duda, Raphael Gasper, Vincent Olieric, Eckhard Hofmann, Anja Hemschemeier

**Affiliations:** 1 Faculty of Biology and Biotechnology, Photobiotechnology, Ruhr University Bochum, Universitätsstr. 150, 44801 Bochum, Germany; 2 Present Address: Charles River Laboratories, Cologne, Germany; 3 Faculty of Biology and Biotechnology, Protein Crystallography, Ruhr University Bochum, Universitätsstr. 150, 44801 Bochum, Germany; 4 Present Address: Max Planck Institute of Molecular Physiology, Otto-Hahn-Str. 11, 44227 Dortmund, Germany; 5 Swiss Light Source (SLS), Paul-Scherrer-Institute (PSI), 5232 Villigen, Switzerland

**Keywords:** Truncated hemoglobin, Nitrite reduction, Oligomerization, Pentacoordination, Ligand tunnels

## Abstract

Hemoglobins (Hbs) utilize heme *b* as a cofactor and are found in all kingdoms of life. The current knowledge reveals an enormous variability of Hb primary sequences, resulting in topological, biochemical and physiological individuality. As Hbs appear to modulate their reactivities through specific combinations of structural features, predicting the characteristics of a given Hb is still hardly possible. The unicellular green alga *Chlamydomonas reinhardtii* contains 12 genes encoding diverse Hbs of the truncated lineage, several of which possess extended N- or C-termini of unknown function. Studies on some of the *Chlamydomonas* Hbs revealed yet unpredictable structural and biochemical variations, which, along with a different expression of their genes, suggest diverse physiological roles. *Chlamydomonas* thus represents a promising system to analyze the diversification of Hb structure, biochemistry and physiology. Here, we report the crystal structure, resolved to 1.75 Å, of the heme-binding domain of cyanomet THB11 (Cre16.g662750), one of the pentacoordinate algal Hbs, which offer a free Fe-coordination site in the reduced state. The overall fold of THB11 is conserved, but individual features such as a kink in helix E, a tilted heme plane and a clustering of methionine residues at a putative tunnel exit appear to be unique. Both N- and C-termini promote the formation of oligomer mixtures, and the absence of the C terminus results in reduced nitrite reduction rates. This work widens the structural and biochemical knowledge on the 2/2Hb family and suggests that the N- and C-terminal extensions of the *Chlamydomonas* 2/2Hbs modulate their reactivity by intermolecular interactions.

**Electronic supplementary material:**

The online version of this article (10.1007/s00775-020-01759-2) contains supplementary material, which is available to authorized users.

## Introduction

Hemoglobins (Hbs) are iron-protoporphyrin-IX (heme *b*)-binding proteins that are found in all kingdoms of life [1]. They arose early in evolution, and it has been proposed that their primordial functions were those of enzymes or sensors of molecular oxygen (O_2_) [2, 3]. In all functional globins, an invariant His residue forms a coordinate bond to the central Fe atom at the proximal site of the heme cofactor, whereas substrates bind to the sixth coordination site of the Fe ion, located at the distal site [4]. Hbs known to date belong to either of two structural sub-classes and are phylogenetically divided into three large families. So-termed 3-on-3 Hbs (3/3Hbs) are built from eight alpha helices, labeled A–H according to their sequential order in animal myoglobin (Mb) and red blood cell hemoglobin (RBC-Hb) [5]. These globins feature a ‘sandwich’ of helices A, E, and F over helices B, G and H. The rather recently discovered so-termed truncated hemoglobins (THBs) instead feature a 2-on-2-fold, in which helices B and E lie over helices G and H [6].

3/3Hbs form two families whose globin domains cluster into those of the Mb-like flavohemoglobins (FHbs) and the globin-coupled sensors (GCSs) [2]. FHbs and GCSs are chimeric proteins consisting of a globin domain and flavin adenine dinucleotide- and nicotinamide adenine dinucleotide-binding domains in the case of FHbs [7] or regulatory output domains in the case of GCSs [8]. Both families contain members that only consist of the globin domain, the FHb single-domain globins, and the GCS family protoglobins and sensor single-domain globins [2, 9, 10]. Metazoan and plant symbiotic and nonsymbiotic 3/3Hbs belong to the single-domain globins within the FHb family [2, 11].

Hbs featuring the 2-on-2-fold, referred to as 2/2Hbs in the following, constitute the third Hb family, and are further subdivided into three phylogenetic groups termed group I, II and III, or N, O and P [12, 13]. A small additional group IV (or Q) has recently been suggested [14]. Originally, 2/2Hbs were described as single-domain globins [15]. Increasingly available genomic data, however, revealed 2/2Hb domains fused to domains with predictable functions, such as protein kinase or nitrate reductase, or to extended N- or C-termini with unknown function, as well as proteins containing several 2/2Hb domains on one polypeptide [16–19].

The ubiquity of globins and the diversity of their primary sequences reflect their diverse functions, of which the well-studied storage and transport of O_2_ is suggested to be a rather recent evolutionary invention. Hbs are known today to bind additional ligands such as hydrogen sulfide, and to catalyze enzymatic reactions, such as the O_2_-dependent nitric oxide (NO) conversion to nitrate, through a reaction termed NO dioxygenation, or nitrite reduction to NO [3, 4]. Although most Hbs studied in vitro can bind various ligands, such as O_2_, NO, carbon monoxide (CO) or cyanide (CN^–^), and perform diverse enzymatic reactions, individual proteins are tuned towards certain functions through variations of the heme-Fe reactivity or its accessibility, modulated by the protein. Tunnels and cavities that allow ligand migration are the first hurdle for a substrate to take [20], while they also allow the accumulation and storage of ligands [21]. Heme accessibility can also be steered by the presence of a distal ligand, which has to be displaced by the exogenous substrate. This can be another small molecule such as water [22] or a distal side residue of the protein in so-termed hexacoordinate globins [23]. The dissociation of a ligand is mostly related to the strength of the bond between the heme-Fe and the substrate, which can be influenced by proximal and distal effects. Proximal effects mainly work through the coordinating HisF8 residue, which affects the electronic structure and the mobility of the heme-Fe [4]. The distal side exerts influence on substrate binding mostly by the presence or absence of H-bond networks that stabilize the ligand [24, 25].

Based on the known influence of specific amino acid side chains, functional trends of Hbs can be predicted to a certain degree by inspecting the primary sequences [e.g. 14, 26]. However, even conserved sequences can fold quite differently and so far, three-dimensional structures have been unpredictable as exemplified by the 2/2Hbs from the unicellular green alga *Chlamydomonas reinhardtii* (*Chlamydomonas* in the following). The microalga contains at least 12 genes coding for class I 2/2Hbs (THB1-12) [16, 27, 28], and the encoded proteins are quite diverse. Whereas THB1, THB2 and THB4 represent single-domain Hbs [29, 30], THB3 and THB5–THB12 feature extended N- and/or C-termini of so far unknown function (note that only THB1 to THB4 have assigned names in the *Chlamydomonas* genome annotation at Phytozome 12; the nomenclature of THB5 to THB12 follows that in Hemschemeier et al. [27]). THB3 lacks the conserved proximal HisF8 residue, THB5 has four consecutive 2/2Hb domains and THB7 features a split globin domain [28, 29, 31]. The proteins that have been analyzed (mostly) in their recombinant forms (THB1–THB4, THB7, THB8, THB10, THB11) show different biochemical and biophysical properties [27, 29–35], suggesting that they fulfil diverse physiological functions.

THB1, THB2 and THB4 are hexacoordinate Hbs and employ LysE10 as the sixth heme-Fe ligand in both the ferric (heme-Fe^III^) and ferrous (heme-Fe^II^) states [29, 30, 32, 33]. LysE10 is conserved in *Chlamydomonas* THB10 and THB11, whose electronic absorption spectra indicate hexacoordination in the ferric state, but whose reduced (heme-Fe^II^) forms are pentacoordinate according to UV–Vis spectroscopic and, in case of THB11, extended X-ray absorption fine structure (EXAFS) analyses [31]. The comparison of the structures of THB1 and THB4 and an analysis of primary sequence patterns suggested to determine hexacoordination in previous studies revealed no clear determinants of LysE10 hexacoordination so far [29]. Notably, a recent study of *Chlamydomonas* THB1 variants, in which LysE10 was exchanged by other residues, indicates that THB1 has an intrinsic structure favoring heme-Fe bis-coordination [36].

THB10 and THB11 differ from the both ferric and ferrous hexacoordinate *Chlamydomonas* THB1, -2 and -4 by possessing moderately extended N-termini, and long (312 and 225 amino acids, respectively) C-termini, which might play a role in determining the heme-Fe ligand set. However, recombinant forms lacking these C-termini still show patterns typical for ferrous pentacoordinate Hbs and overall very similar UV–Vis spectroscopic and EXAFS features compared to the full-length proteins [31]. Clearly, more structural data are required to understand the biochemical and, eventually, functional diversification of 2/2Hbs.

Here, we report the first crystal structure of a *Chlamydomonas* pentacoordinate 2/2Hb, the heme-binding domain (HBD) of *Chlamydomonas* THB11 in its cyanomet form. The tertiary structure recapitulates the typical 2/2Hb fold, but unusual features are observed, such as a kink in helix E, a tilted heme plane and a tunnel system possibly shielded by Met residues. While our aim was to obtain the structure of the full-length protein including its long C-terminus, we were unable to obtain crystals of any protein variant extending the actual globin domain. As this suggested these protein parts to be flexible or disordered, we analyzed variably truncated THB11 forms and found that both N- and C-termini prompted oligomerization, whereas the HBD was monomeric. Notably, C-terminal truncations resulted in reduced nitrite reduction rates.

## Experimental procedures

### Production of recombinant THB proteins

#### Generation of constructs

The *C. reinhardtii* 2/2Hbs encoded by transcripts *Cre14.g615400.t1.2* (THB1) and *Cre16.g662750.t1.2* (THB11) were produced in recombinant form as reported before by Huwald et al. [31] (see Tab. S1 and S2 in the supplementary material for details). Length variants of THB11 were generated that lacked the polypeptide extensions N- and C-terminal of the globin domain. These are labeled by the suffices -N (N-terminal truncation), -C (C-terminal truncation) or -NC (both termini truncated). THB11-C has been described before [31]. Expression vectors were purchased from IBA Lifesciences (https://www.iba-lifesciences.com), namely pASK-IBA3plus for sequences encoding THB1, THB11 and THB11-C, and pASK-IBA5plus for THB11-N and THB11-NC (Tab. S1 in the supplementary material). Sequences expressed from pASK-IBA3plus and pASK-IBA5plus result in proteins equipped with a C-terminal or N-terminal *Strep*-tag II, respectively. Sequences for pASK-IBA5plus were designed in a way that the *Strep*-tag II could be cleaved off using TEV protease (Tab. S1, Tab. S2 in the supplementary material). The sequence coding for THB1 was codon-adapted for *Escherichia coli* K12 and provided by Eurofins MWG GmbH (https://www.eurofins.de), equipped with *Bsa*I recognition sites required for cloning. The DNA sequence is provided in Huwald et al. [31]. The THB11 encoding sequences were amplified from cDNA obtained from the *C. reinhardtii* wild-type CC-124 mt-[137c] (Chlamydomonas Resource Center, University of Minnesota, MN, USA) as described in Huwald et al. [31], utilizing the oligonucleotides listed in Tab. S2 in the supplementary material. All sequences were cut by *Bsa*I and ligated with *Bsa*I-cut expression vectors. Cloning was done using *E. coli* strain DH5α MCR grown in LB Broth (Lennox) or on LB Agar (Lennox) from Carl Roth GmbH (https://www.carlroth.com) supplied with 100 µg × ml^−1^ ampicillin at 37 °C. Liquid cultures ware shaken at 180 rpm. The expression constructs were sequenced by the DNA sequencing service at the chair for biochemistry, Biochemistry I, receptor biochemistry, at the Ruhr University Bochum, Germany.

#### Heterologous protein production in *E. coli*

Recombinant protein production was done in *E. coli* strain Rosetta™ (DE3) (F^−^*ompT hsdS*_B_ (r_B_^−^ m_B_^−^) *gal dcm* (DE3) pRARE (Cam^R^) from Merck (https://www.merckmillipore.com). Cells were transformed through electroporation using 100 ng of the respective expression vector and grown overnight at 37 °C in liquid LB medium containing 100 µg × ml^−1^ ampicillin and 25 µg × ml^−1^ chloramphenicol. For heterologous protein production, 5 ml of the precultures were transferred to 500 ml Terrific Broth (TB) medium (24 g × l^−1^ yeast extract, 12 g × l^−1^ tryptone, 4 ml × l^−1^ glycerol, 0.017 M KH_2_PO_4_, 0.072 M K_2_HPO_4_) with 100 µg × ml^−1^ ampicillin and 25 µg × ml^−1^ chloramphenicol in 2 l Erlenmeyer flasks. In case of all constructs, the *E. coli* cultures were shaken at 180 rpm and expression was induced by adding 200 ng × ml^−1^ anhydrotetracycline (AHT) (IBA Lifesciences), while growth temperature, the OD_600_ at which AHT was added and the expression time were optimized for each protein (Tab. S2 in the supplementary material). Afterwards, the cells were harvested by centrifugation [4 °C, 6200×*g*, 20 min) and washed with 50 ml buffer W (100 mM Tris–HCl pH 8.0, 150 mM NaCl, 2.5 mM ethylenediaminetetraacetic acid (EDTA)]. Sometimes the cell pellets were frozen at − 20 °C before protein purification.

### Purification of THBs

Recombinant 2/2Hbs were purified by *Strep*-tag affinity chromatography and additionally by anion exchange chromatography (AEC) and size exclusion chromatography (SEC) when indicated. In all cases, elution fractions were concentrated employing Amicon^®^ Ultra-4 Centrifugal Filter Units, 10 K (Merck), and the identity and purity of the proteins were analyzed by sodium dodecyl sulfate polyacrylamide gel electrophoresis (SDS-PAGE) followed by Coomassie-Blue R250 staining according to Laemmli [37]. If not directly used for experiments, protein solutions were snap-frozen in liquid nitrogen and stored at − 80 °C.

#### *Strep*-tag affinity chromatography

After heterologous expression of the 2/2Hb encoding sequences, the *E. coli* cell pellets were resuspended in 20 ml buffer W and lysed by sonication on ice (six cycles, 25 s each, output energy of 50%) using a Branson Sonifier 250 (Branson Ultrasonics Corporation, https://www.emersonindustrial.com/en-US/branson/). Insoluble components were removed by ultracentrifugation (4 °C, 180,000×*g*, 45 min) and subsequent passing of the supernatant through a 0.2 µm pore size sterile filter. The cleared lysate was applied to a gravity column containing 2 ml of Strep-Tactin^®^ Superflow^®^ high-capacity resin (IBA Lifesciences), equilibrated with buffer W. The column was washed with 10 ml buffer W, and then the bound proteins were eluted applying 10 ml buffer E (buffer W containing 2.5 mM desthiobiotin). Recombinant THB1 was judged to be pure after the affinity chromatography step. The remaining proteins were further purified through AEC and SEC.

#### AEC

AEC and SEC (see below) were performed utilizing an Äkta HPLC system and columns from GE Healthcare Life Sciences (https://www.gelifesciences.com). A HiTrap Q HP 5 ml column was used for AEC. The buffers of the protein solutions were first exchanged to 25 mM Tris–HCl pH 8.0 employing PD MidiTrap G-25 columns (GE Healthcare Life Sciences). The same buffer was used for equilibrating the column as well as for washing. After washing, proteins were eluted by a linear salt gradient established using the equilibration/wash buffer supplemented with 1 M NaCl. The absorbance at *λ* = 280 nm was recorded to collect fractions that contained protein.

#### SEC

Preparatory SEC was performed using a HiLoad Superdex 16/600 200 pg column and buffer W as the equilibration and running buffer. Elution fractions that contained protein as indicated by the absorbance at *λ* = 280 nm were collected. Calibration of the column was achieved utilizing the Gel Filtration Markers Kit for Protein Molecular Weights 29,000–700,000 Da (Sigma-Aldrich; https://www.sigmaaldrich.com).

#### Cleavage of proteins using TEV protease

TEV protease was produced and purified according to Tropea et al. [38]. For crystallization, the N-terminal *Strep*-tag of THB11-NC was removed after the AEC step. 1 mg of TEV protease was incubated with 10 mg of target protein in the presence of 1 mM dithiothreitol (DTT) and 0.5 mM EDTA at 4 °C overnight. The TEV protease was removed by loading the mixture onto a gravity-flow column containing cOmplete™ His-Tag Purification Resin (Sigma-Aldrich/Roche Diagnostics, https://www.roche-diagnostics.ch/) and washing off the target protein using a 100 mM Tris–HCl buffer, pH 8.0, 150 mM NaCl. The wash fraction was concentrated and purified by SEC (see above).

### Analysis of protein oligomeric states

Protein oligomerization states were investigated by analytical SEC and native PAGE. SEC was performed as described above, except that a Superdex 200 Increase 10/300 GL column was used. This column was calibrated using ribonuclease, carbonic anhydrase, ovalbumin, conalbumin, aldolase and ferritin from the Gel Filtration Calibration Kits LMW and HMW from GE Healthcare. Native PAGE was conducted according to the protocol for SDS-PAGE [37], except that SDS and β-mercaptoethanol (β-ME) were omitted from the gels and buffers, and the protein samples were not heated. Bands on the native gels were visualized employing zinc/imidazole staining [39]. Afterwards, the bands were cut from the gels and incubated at 4 °C overnight in 30 µl SDS-PAGE sample buffer (50 mM Tris–HCl pH 6.8, 2.5% (w/v) SDS, 5% (v/v) glycerol, 2.5% (v/v) β-ME, 0.01% (w/v) Bromophenol Blue). The samples were centrifuged and the supernatants were transferred to fresh tubes and analyzed by SDS-PAGE.

### UV–Vis spectroscopic analyses and determination of nitrite reduction rates

Steady-state UV–Vis spectra were recorded from *λ* = 250 to *λ* = 800 nm using the UV-2450 spectrophotometer from Shimadzu (https://www.shimadzu.com/) at a resolution of 1 nm, a gap width of 2 nm and at a fast sampling rate. Recombinant 2/2Hbs at a concentration of 5–10 µM heme in 700 µl buffer W were prepared in UV-cuvettes micro (BRAND; https://www.brand.de) in an anoxic tent with a nitrogen atmosphere containing ca. 1% molecular hydrogen. All buffers and solutions were also kept under anoxic conditions. Before the cuvettes were taken out of the tent they were sealed with white rubber Suba-Seal^®^ 21 (Sigma-Aldrich). Horse heart Mb (Sigma-Aldrich) (10 µM) was included in all experiments as a control for the set-ups. All samples were kept on ice until just before the UV–Vis spectra were recorded. The heme groups of the proteins were reduced by adding sodium dithionite (NaDt) to a final concentration of 200 µM, and oxidized in the presence of 50 µM potassium ferricyanide. For O_2_-binding assays, excess NaDt was removed by exchanging the buffer using PD MidiTrap G-25 columns and the cuvette was opened to air. NO binding was analyzed by adding diethylamine NONOate diethylammonium salt (DEA-NONOate; Sigma-Aldrich). A 10 mM solution in buffer W was freshly prepared for each experiment under anoxic conditions and added to a final concentration of 200 µM.

Nitrite reduction rates were determined UV–Vis spectroscopically following the decrease of the Soret peak maxima of deoxyglobins (heme-Fe^II^) upon the formation of ferrous iron-nitrosyl globins. To determine the nitrite reduction rates of the globins under pseudo-first order conditions, the assays were conducted in the presence of NaDt. The deoxy forms were prepared in cuvettes in an anoxic tent on ice as described above. Globin concentrations were adjusted to 5 µM heme in 600 µl of anoxic 50 mM HEPES buffer, pH 7.4, 100 mM NaCl, supplemented with 600 µM NaDt. UV–Vis spectra of these samples were recorded to obtain the initial spectrum for each protein in its deoxy state. Then, anoxic potassium nitrite dissolved in the same buffer was added to the samples to reach final concentrations of 0.25, 0.5, 0.75 and 1 mM using a gas-tight syringe (Hamilton; https://www.hamiltoncompany.com/). The cuvettes were gently shaken and then immediately placed into the UV–Vis spectrophotometer at 20 °C. Absorption at the Soret peak maxima of deoxy Hbs was measured every 3 s until the absorption stayed constant, which usually took about 10 min. Final spectra were recorded to ensure that formation of the ferrous nitrosyl complex had occurred. Each experiment was done in technical triplicates per nitrite concentration, and from at least two independently produced batches of recombinant proteins. The pseudo first order rate constants *k*_obs_ were obtained in OriginPro from exponential decay fits of the absorption changes in time, and averaged per experiment. Linear fits of the *k*_obs_ mean values obtained using different nitrite concentrations yielded the bimolecular reaction rates.

### Crystallization, structure determination and structure analysis

THB11-NC was produced and purified as described above. After the removal of the *Strep*-tag using TEV protease and a final SEC, the heme group was oxidized applying a ten-fold excess of ferricyanide, which was removed afterwards using PD MidiTrap G-25 columns. The cyanomet (heme-Fe^III^-CN^–^) form was then generated by adding a two-fold excess of potassium cyanide (KCN). Screening for crystallization conditions was done applying the sitting drop vapor diffusion approach, utilizing Crystal Phoenix dispenser (Art Robbins Instruments, https://www.artrobbins.com). Mixtures of the protein and precipitant solutions (100 nl:100 nl) were incubated in sitting-drop vapor diffusion plates (Corning^®^ 3550 plates, https://www.corning.com) at 4 °C or 18 °C. Initial crystals were obtained at 1:1 mixtures of protein (19 mg × ml^−1^ in 15 mM Tris–HCl pH 8.0) with reservoir solutions containing 0.1 M HEPES (pH 6.5–7.5) and 20–30% PEG (6.000–8.000) incubated at 4 °C. Final crystals appeared when mixing 1 µl protein solution (19 mg × ml^−1^ in 20 mM HEPES, pH 7.0) with 1 µl 0.1 M HEPES pH 7.0 and 21% PEG 6.000 on CrystalClear D Strips (Douglas Instruments, Berkshire, UK) at 4 °C. The crystal was first transferred to a cryoprotectant solution (0.1 M HEPES pH 7.0, 30% (w/v) PEG 8.000) using a micro loop and then snap-frozen in liquid nitrogen. Single-wavelength Anomalous Dispersion (SAD) datasets at the Fe K-edge were collected at beamline X06DA-PXIII (https://www.psi.ch/sls/pxiii) at the Swiss Light Source (SLS, Villigen PSI, Switzerland), applying five 360° sweeps at different chi angles using the PRIGo goniometer [40]. Processing and scaling of diffraction data were done using *XDS* and *XSCALE* [41]. *SHELX* [42] within *HKL2MAP* [43] was used to identify the position of the Fe ion. *PHENIX.PHASER* and *PHENIX.AUTOBUILD* [44] were applied to generate the electron density map and the first atomic model based on the primary sequence of THB11-NC. The model was refined using *PHENIX.REFINE* [44, 45] and manually build using *COOT* [46]. Tunnels were calculated employing the CAVER 3.0.1 PyMOL plug-in [47] with default settings (minimum probe radius: 0.9, shell depth: 4, shell radius: 3), selecting the heme-Fe as starting point and excluding the CN^–^ ligand. Heme distortions were analyzed by Normal-coordinate Structural Decomposition [48, 49] (https://mliptak.w3.uvm.edu/nsd.html), and structural homologues were identified using the heuristic PDB search on the DALI server [50] (http://ekhidna2.biocenter.helsinki.fi/dali/). Multiple primary sequence alignments were done using Clustal Omega [51] (https://www.ebi.ac.uk/Tools/msa/clustalo/). First, the full-length sequences were aligned in order to determine the HBD boundaries. For the final alignment, only the HBD sequences were used as input.

## Results

The aim of this study was to obtain structural information on a ferrous pentacoordinate *Chlamydomonas* 2/2Hb, THB11 (gene ID Cre16.g662750), which is one of the algal Hbs that feature extended N- and, particularly, C-termini of yet unknown function. To investigate the role(s) of these termini, we created recombinant length variants of THB11 lacking either one or both termini, namely THB11-N, THB11-C and THB11-NC, lacking the N-terminal 41 amino acids (-N), the C-terminal 225 residues (-C) or both (-NC). We have compared THB11 and THB11-C before, and the truncation of the C terminus hardly affects steady-state ligand binding and the geometry of the active site as studied by UV–Vis spectroscopic and EXAFS analyses [31]. THB11-N and THB11-NC also bound heme stably, and the UV–Vis spectra of the oxidized (Fe^III^), reduced (Fe^II^), oxy (Fe^II^–O_2_) and ferrous nitrosyl (Fe^II^–NO) forms were very similar to those reported before for THB11 and THB11-C [31] (Fig. S1 in the supplementary material). We attempted to crystallize all THB11 length variants, however, despite extended screenings for suitable crystallization conditions, only THB11-NC, which is reduced to the heme-binding domain (HBD), and from which the *Strep*-tag had been removed, formed crystals.

### The THB11-NC tertiary structure recapitulates the typical 2/2Hb fold

THB11-NC crystallized in space group *P*2_1_ 2_1_ 2_1_, with one molecule in the crystallographic asymmetric unit. The structure could be refined to a resolution of 1.75 Å (Table 1) (PDB entry: 6TD7). Eleven N-terminal (GSAGTSTATNA; note that the residues GSA are part of the TEV cleavage site) and five C-terminal (AAEEA) amino acid residues could not be resolved. Six residues (Glu29, Arg54, Ser58, Leu109, Met123 and Leu124; the numbering follows the sequence of the polypeptide whose structure was resolved) were modeled to be present in two conformations. Both the heme cofactor and the CN^–^ ligand could be placed in clear electron density (Fig. S2 in the supplementary material).

**Table 1:** Data collection, processing and refinement statistics

Data collection	
Beamline	SLS-X06DA
No. of scaled sweeps	5
Resolution (Å)	38.54–1.75 (1.81–1.75)
Cell parameters	
*a*, *b*, *c* (Å)	31.04, 42.62, 90.26
*α, β, γ* (°)	90, 90, 90
Space group	P2_1_ 2_1_ 2_1_
Wavelength (Å)	1.739
No. of observations	594,012 (4,429)
Anomalous pairs merged	
No. of unique reflections	11,933 (711)
Completeness (%)	94.3 (58.4)
Multiplicity	49.8 (6.2)
Average *I/σ I*	38.4 (2.6)
CC (1/2)	1.0 (0.82)
*R*_merge_	9.5 (56.6)
*R*_meas_	9.6 (61.7)
Anomalous pairs unmerged	
No. of unique reflections	21,945 (711)
Completeness (%)	94.3 (58.4)
Multiplicity	27.1 (3.3)
Average *I/σ I*	28.4 (1.9)
CC (1/2)	1.0 (0.73)
*R*_merge_	8.8 (52.6)
*R*_meas_	8.9 (61.9)
Wilson B-factor (Å^2^)	17.99
Phasing	
Resolution for phasing (Å)	1.75
Iron sites	1
Figure of merit	0.35
Refinement	
*R*_work_/*R*_free_	0.173 / 0.210
Root-mean-square deviation bond length (Å)	0.005
Root mean square deviation angles (°)	0.893
Ramachandran favored (%)	99
Ramachandran outlier (%)	0
Average B‐factor (Å^2^)	18.9
Protein (Å^2^)	17.6
Ligand (Å^2^)	19.3
Solvent (Å^2^)	26.9
No. of atoms (without riding hydrogens)	1,260
Protein	1,062
Ligands	45
Water	153
PDB code	6TD7

Values in parenthesis represent the data for the highest resolution shell

*SLS* Swiss Light Source

**Fig. 1:**
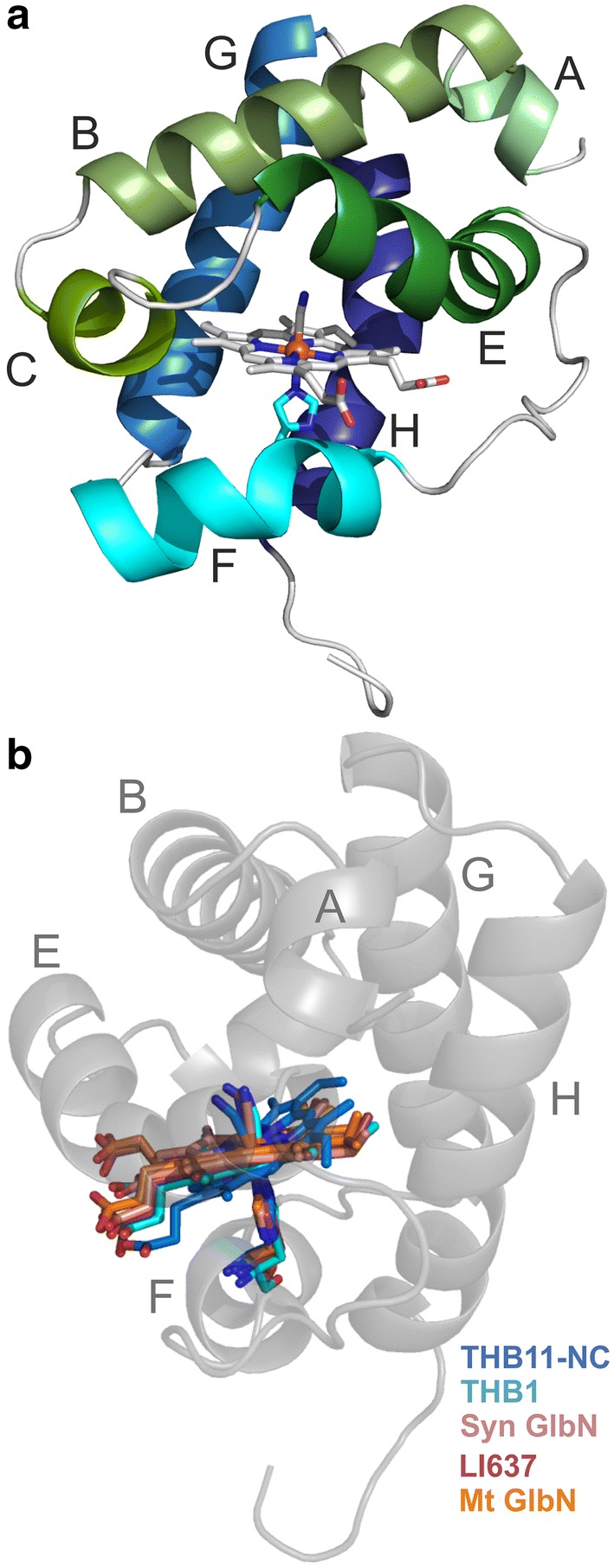
The crystal structure of the THB11 HBD reveals the typical class I 2/2Hb structure, but a kink in helix E and a tilt of the heme group. **a **Cartoon diagram of THB11-NC (PDB: 6TD7). Helices are colored individually and labeled according to the Perutz nomenclature [5]. **b** The structures of the indicated cyanomet 2/2Hbs (THB1: *C. reinhardtii* THB1, PDB: 6CII; Syn GlbN: *Synechocystis* 2/2Hb, PDB: 1S69 chain A; LI637: *C. eugametos* 2/2Hb LI637, PDB: 1DLY chain A; Mt GlbN: *M. tuberculosis* 2/2Hb, PDB: 1RTE chain B) were aligned to THB11-NC. The view is from the side of the A helix and the EF-turn, respectively. The heme groups, coordinating HisF8 residues and CN^–^ ligands are shown for all proteins. For orientation, the tertiary structure of THB11-NC is depicted in transparent gray. The figures were generated in PyMOL

The tertiary structure of the THB11 HBD is mostly typical for group I 2/2Hbs [6, 25]. It features two antiparallel helix pairs, BE and GH, surrounding the heme cofactor, a short A- and an absent D-helix, and a long EF-loop (Fig. 1a). The four best-matching structures (*Z* scores of 17.7, 16.9, 16.8 and 15.6) of globins in the cyanomet form detected by the DALI server [50] were THB1 from *Chlamydomonas* [32, 36] (PDB 6CII), cyanoglobin (GlbN) from *Synechocystis* PCC 6803 [52] (PDB 1S69), LI637 from *Chlamydomonas eugametos*/*moewusii* [6] (PDB 1DLY) and GlbN from *Mycobacterium tuberculosis* [53] (PDB 1RTE). The higher structural similarity of THB11-NC to THB1 and cyanoglobin is reflected in the F-helix, which is comprised of only one helical turn in the 2/2Hbs from *C. eugametos* and *M. tuberculosis*, but of about three turns in the former three proteins (Fig. S3 in the supplementary material). THB11-NC differs from other class I 2/2Hb structures by featuring a noticeable kink in the E-helix (Figs. 1a, S3).

### The active site displays a moderate ligand-stabilizing H-bond network

**Fig. 2:**
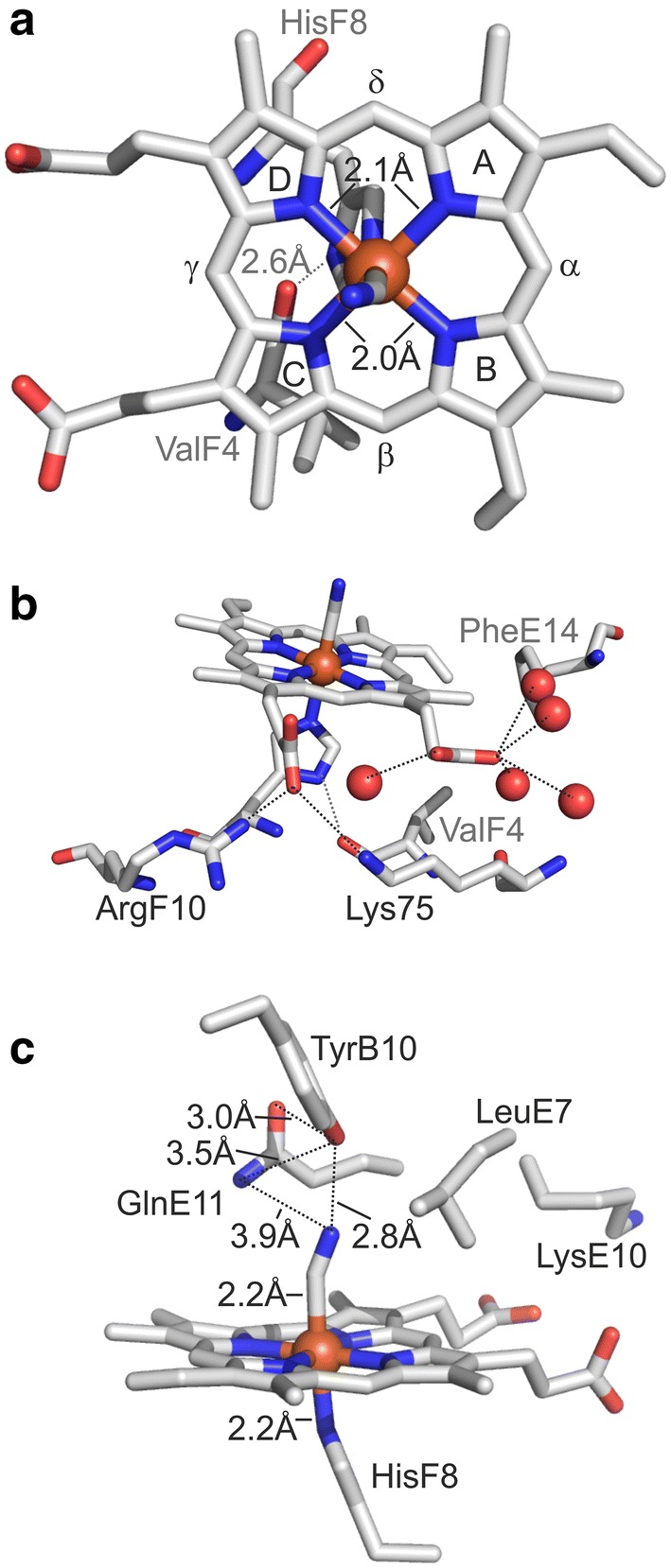
The heme environment of THB11-NC. **a** The top view shows the moderately staggered and tilted imidazole ring of the heme-coordinating HisF8(81) residue and the H-bond distance between the HisF8(81) Nδ-atom and the backbone carbonyl group of ValF4(77). **b** The heme propionates are in polar contact distance to ArgF10(83), Lys75 (EF-loop) and water molecules. The highly conserved heme-shielding PheE14(60) is also shown. **c** The side view shows heme-Fe ligand distances as well as distal site residues reported to play a role in ligand stabilization by H-bond networks in class I 2/2Hbs (B10, E7, E11, represented by Tyr32, Leu53 and Gln57 in THB11-NC) as well as LysE10(56). **a**–**c** Figures were created and distances measured in PyMOL. Putative polar contact distances are indicated by dashed lines

Only one heme orientation isomer could be fitted into the electron density map of THB11-NC, and the porphyrin ring is rotated by 180° along the *α*–*γ* axis compared to those of THB1, LI637 and *Synechocystis* or *M. tuberculosis* GlbN. Compared to those of additional 2/2Hbs, the heme plane is notably tilted when inspecting the THB11 HBD structure from the side of the A-helix (Fig. 1b). Normal-coordinate structural decomposition analysis [48, 49] indicates that the heme group of THB11-NC features an overall out of plane displacement of 0.49 Å, and the heme distortions are dominated by doming and ruffling. The distances between the Fe ion and the porphyrin N-atoms are 2.1 Å (pyrrole rings A and D) and 2.0 Å (pyrrole rings B and C), and 2.2 Å to both the HisF8 Nε atom and the CN^–^ ligand (Fig. 2a, c). In Hbs, viewed from the top, the imidazole plane of HisF8 can lie parallel to the line that connects opposite pyrrole ring N atoms of the porphyrin ring system, or form an angle of about 45°. These positions are referred to as eclipsed and staggered, respectively. The imidazole ring of THB11-NC HisF8(81) (note that residues are indicated in the Perutz nomenclature, followed by their numbers in the structure in brackets) is in a moderately staggered position, and its plane is tilted towards the heme group (Fig. 2a, c). The Nδ-atom of HisF8(81) is in moderate H-bond distance (2.6 Å) to the backbone carbonyl of ValF4(77), while the heme propionates form polar contacts (2.3 Å to 3.3 Å) to ArgF10(83), Lys75 in the EF-loop and five water molecules (Fig. 2a, b).

In group I 2/2Hbs, polar residues at the topological positions B10, E7 and/or E11 have been shown to contribute to ligand stabilizing H-bond networks [25], and references therein] (see an alignment of several *Chlamydomonas* 2/2Hb HBDs to additional 2/2Hbs in Fig. S4 in the supplementary material). In the distal site of THB11-NC, the CN^–^ ligand lies in moderate H-bond distance to the hydroxyl group of TyrB10(32) (2.8 Å), whereas position E7 is occupied by Leu53, and the distance of the ligand to the amide group of GlnE11(57) is 3.9 Å (N atom) or 4.9 Å (O atom). The TyrB10(32) hydroxyl group itself is in moderate to weak H-bond distance to GlnE11(57), namely 3.0 Å (O atom) and 3.5 Å (N atom), respectively (Fig. 2c). LysE10(56) points away from the heme cofactor, being located at the E helix side that is averted from the distal niche.

### The THB11-NC crystal structure indicates an uncommon tunnel system

**Fig. 3:**
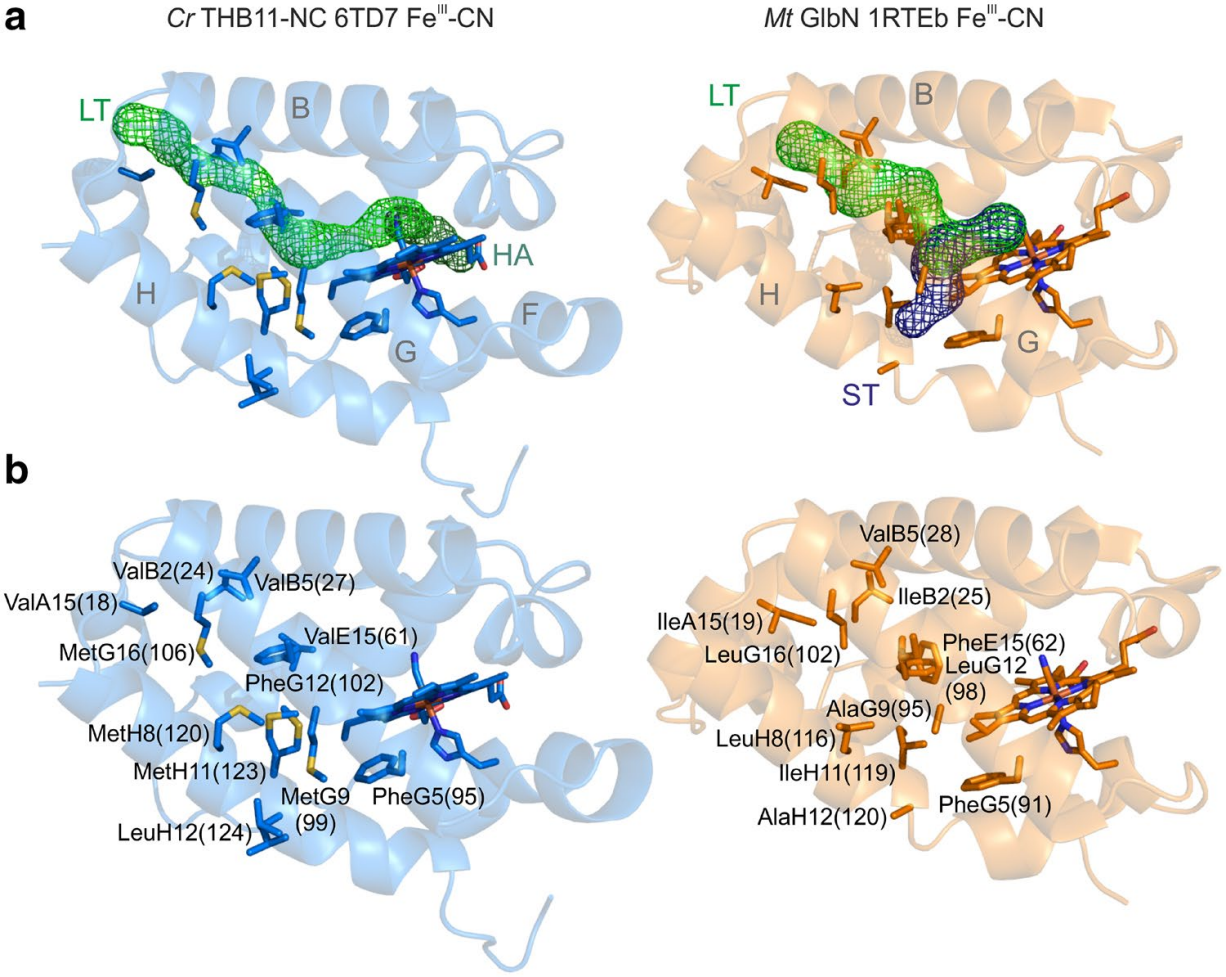
Possible ligand access routes can be identified in THB11-NC. **a** Tunnels that start at the heme-Fe of *Chlamydomonas* (*Cr*) THB11-NC (PDB: 6TD7) and *M. tuberculosis* (*Mt*) GlbN (PDB: 1RTE, chain B) were computed by Caver 3.0.1, employing a minimal probe radius of 0.9, and visualized in PyMOL. Residues reported to line the long (LT) and short tunnels (ST) are represented as sticks and, in panel **b**, are labeled according to the Perutz nomenclature, including their position in the respective sequences. HA: heme access site observed in THB11-NC

Group I 2/2Hbs have protein cavities or tunnels involved in ligand migration and accumulation. In *M. tuberculosis* GlbN, a longer tunnel (LT) is positioned roughly perpendicular to the heme plane and exits the protein between the hinges of the A- and B- and the G- and H-helices. A shorter tunnel (ST) runs approximately parallel to the heme plain and exits the protein between helices G and H [21, 54] (Fig. 3a). Similar L-shaped tunnel systems have been described for additional class I 2/2Hbs, such as that of *Tetrahymena pyriformis* and *C. eugametos* [21, 55]. In *Synechocystis* and *Synechococcus* GlbNs, ligand exchange might also occur at the solvent exposed heme edges [52, 56, 57]. In the *Chlamydomonas* THB11-NC crystal structure, the LT, but not the ST could be detected by Caver, and a tunnel exiting at the solvent exposed heme edge was computed (Fig. 3a).

In *M. tuberculosis* GlbN, the LT opens to the solvent by an aperture formed by IleA15, IleB2, ValB5, and LeuG16, and the ST entry is formed by PheG5, AlaG9, LeuH8, IleH11 and AlaH12 [21, 54]. In THB11-NC, these positions are at similar topological sites. A15, B2 and B5 are occupied by Val residues 18, 24 and 27, whereas Met106 is at the G16 site (Fig. 3b). PheG5 is highly conserved in class I 2/2Hbs [13, 14] (also see Fig. S4). In THB11-NC, a Phe is also found at the respective site (residue 95), however, the G9, H8, H11 and H12 positions are occupied by Met99, Met120, Met123 and Leu124, respectively (Fig. 3b). Met123 as well as Leu124 were modeled to be present in two conformations (63% and 37% occupancy in both cases). The intersection of the LT and ST is formed by residues E15 and G12, which are both Leu residues in *T. pyriformis* TrHbN (Leu54 and Leu90) [55], whereas Phe62 forms the so-termed E15 gate in *M. tuberculosis* GlbN [54, 58, 59]. In THB11-NC, E15 is represented by Val61. Here, however, the position opposite to the E15 residue is occupied by a bulky Phe residue (PheG12(102)) (Fig. 3b).

### The N- and C-terminal extensions of THB11 promote oligomerization

**Fig. 4:**
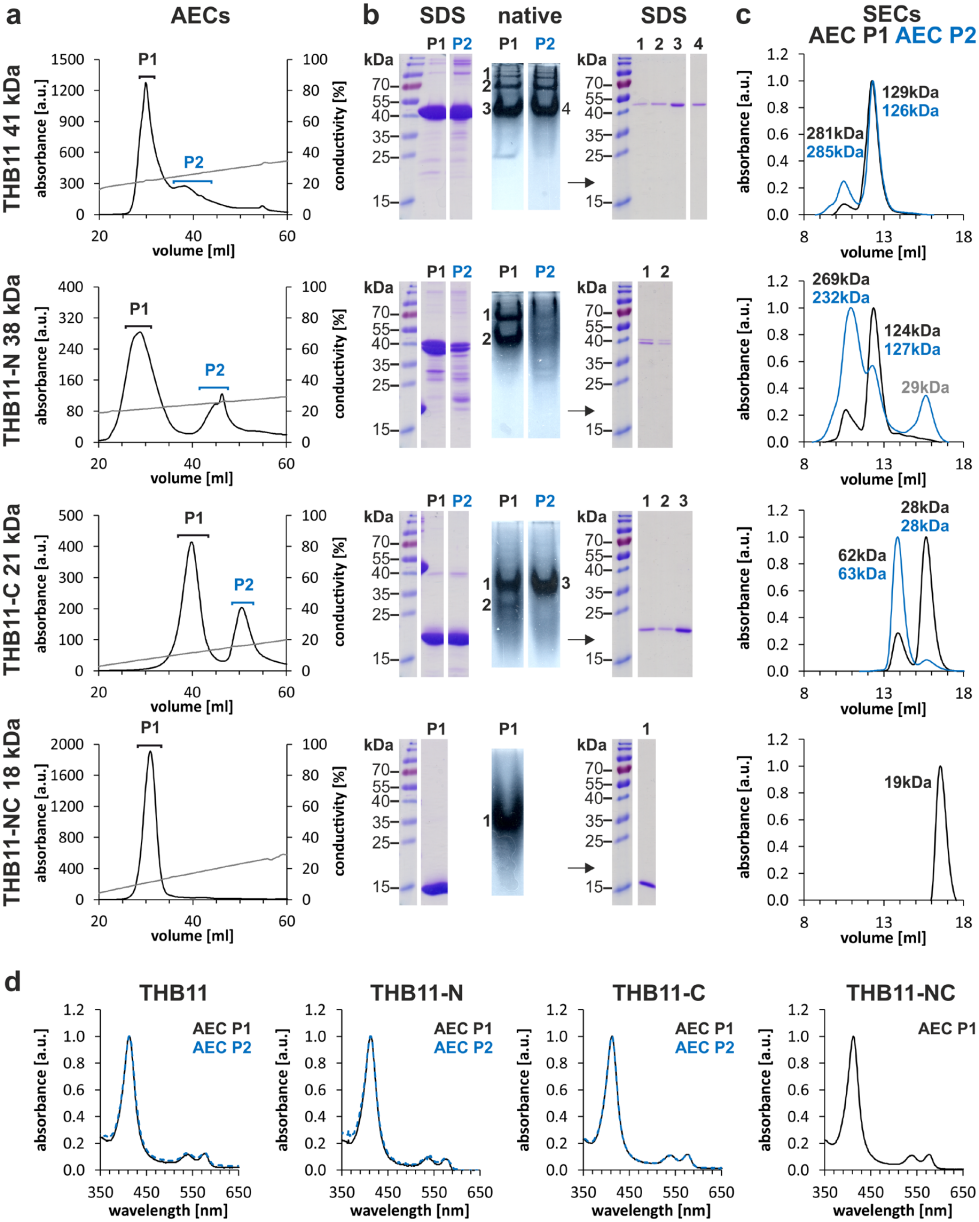
THB11 length variants form different oligomeric states. **a** Anion exchange chromatography (AEC) profiles of the indicated protein variants, which had been purified from *E. coli* cell lysates by *Strep*-tag affinity chromatography before. The brackets above the peaks indicate the elution fractions that were combined for subsequent analyses [black, blue: peak (P) 1, 2]. The molecular mass of each protein calculated from the primary sequence are indicated to the left. **b** Denaturing (SDS) and native gel electrophoreses of the protein solutions collected from the AEC peaks P1 and P2. The numbered bands observed in the native gels were cut out and subsequently subjected to denaturing gel electrophoresis. **c** Size exclusion chromatography (SEC) profiles of the proteins present in the two AEC peaks. kDa labels within the SEC graphs indicate the molecular masses calculated from the elution volumes according to the column’s calibration curve, colors indicate the original AEC P1 (black) or P2 (blue). **d** UV–Vis spectra of the protein solutions collected from the two AEC peaks, P1 and P2. The spectra were normalized with regard to the Soret maxima (set to 1)

Despite many trials we were unable to obtain crystals of a *Chlamydomonas* THB11 protein variant containing either N- or C-terminal extensions. This suggests that these termini might be flexible, or that they result in structural heterogeneity within the protein population. We noted that the purification of all THB11 length variants except THB11-NC through anion exchange chromatography (AEC) repeatedly resulted in two well defined elution peaks (Fig. 4a), whose dominant proteins represented the respective recombinant THB11 variant according to their size in denaturing gel electrophoresis (Fig. 4b). The protein solutions from both AEC peak fractions were subsequently tested for possible oligomeric states by native gel electrophoresis and size exclusion chromatography (SEC). All THB11 length variants except THB11-NC were present in more than one band after native gel electrophoresis, whereas the number of bands differed between the variants as well as the two AEC fractions, and was highest in case of full-length THB11 (Fig. 4b). Analytical SEC showed that all protein variants except THB11-NC eluted as two major protein peaks (Fig. 4c). According to the calibration of the SEC column, these represented approximately tri- and heptamers in case of THB11 and THB11-N, and mono- and trimers in case of THB11-C, whereas the elution volume of the single THB11-NC peak corresponded to a monomer. The protein solutions obtained from the two AEC elution fractions were represented by different ratios of the two oligomeric states, which was particularly pronounced in case of THB11-C (Fig. 4c). UV–Vis spectra of the THB11 length variants present in the different AEC peaks were almost identical in all cases and represented the oxy forms (Fe^II^–O_2_) (Fig. 4d).

### The THB11 C terminus influences nitrite reduction activities

**Fig. 5:**
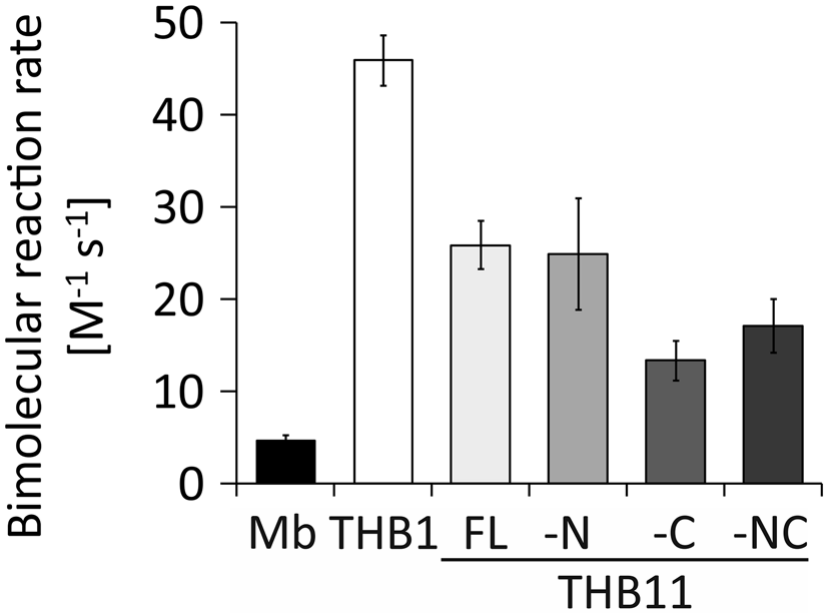
Nitrite reduction rates of THB11 length variants. Recombinant deoxy *Chlamydomonas* THB11 length variants as well as horse heart Mb and *Chlamydomonas* THB1 at concentrations of 5 µM heme were incubated in anoxic 50 mM HEPES buffer, pH 7.4, 100 mM NaCl, supplemented with 600 µM NaDt and 0.25 to 1 mM potassium nitrite. The decrease of the Soret peak maxima of deoxy (heme-Fe^II^) globins was followed spectroscopically at 20 °C. Pseudo first order rate constants *k*_obs_ were obtained from exponential decay fits of the absorption changes in time (see Fig. S5). Linear fits of the *k*_obs_ mean values obtained at different nitrite concentrations yielded the bimolecular reaction rates. Experiments were done in technical triplicates per nitrite concentration from at least two independent protein batches. The bars indicate the averages of reaction rates calculated per experiment, and error bars indicate the standard deviation. *FL* full length, *-N* without N terminus, *-C* without C terminus, *-NC* without N- and C-termini

As noted before for THB11 and THB11-C [31] and shown here also for THB11-N and THB11-NC (Fig. S1 in the supplementary material, Fig. 4d), steady-state UV–Vis spectra of the length variants in different states and in the presence or absence of ligands are very similar. To test whether the termini of THB11 exert an influence on the kinetics of a reaction, we determined the nitrite reduction rates of the protein variants. Nitrite reduction to NO by the deoxy (heme-Fe^II^) forms of globins [60] has been suggested to be a potential in vivo function especially of globins from photosynthetic organisms [61, 62] (also see discussion), and the kinetics of this reaction can be traced robustly by UV–Vis spectroscopy. The reaction can be described as Hb-Fe^II^ + NO_2_^–^ + H^+^  → Hb-Fe^III^ + NO + OH^–^, followed by a rapid binding of NO to additional deoxyglobins (Hb-Fe^II^ + NO → Hb-Fe^II^−NO). Thereby, in the absence of reducing agents, two deoxyglobins react with nitrite to one iron-nitrosyl globin (Hb-Fe^II^−NO) and one metglobin (Hb-Fe^III^), whereas in the presence of a reductant such as NaDt, Hb-Fe^III^ is rapidly re-reduced and deoxyglobin is completely converted to Hb-Fe^II^−NO [63]. Here, nitrite reduction rates were thus analyzed by incubating the deoxy (Fe^II^) forms with varying concentrations of nitrite in the presence of excess reductant (600 µM NaDt), resulting in pseudo-first order reaction kinetics (Fig. S5 in the supplementary material shows exemplary kinetic traces and the averaged pseudo first order rate constants *k*_obs_). Horse heart Mb as well as *Chlamydomonas* recombinant THB1 served as controls, and their nitrite reduction rates of 4.7 ± 0.6 M^−1^ s^−1^ (Mb) and 45.9 ± 2.7 (THB1) (Fig. 5) correlated with reported rates (Mb: e.g. 2.9 M^−1^ s^−1^ [64] or 5.5 M^−1^ s^−1^ [65]), determined at pH 7.4 and pH 7.41, respectively; THB1: 98 M^−1^ s^−1^, determined at pH 6.6 [30]). Full-length THB11 had a nitrite reduction rate of 25.9 ± 2.6 M^−1^ s^−1^, which was similar to the rate observed for THB11-N (24.9 ± 6.1 M^−1^ s^−1^), but higher than those of both C-terminally truncated variants, which were 13.4 ± 2.1 M^−1^ s^−1^ (THB11-C) and 17.1 ± 2.9 M^−1^ s^−1^ (THB11-NC) (Fig. 5).

## Discussion

The characterization of the 12 class I 2/2Hbs of *Chlamydomonas* is at an early stage. The available physiological, transcript level and biochemical data speak for diverse physiological functions and fine-tuned protein characteristics [29, 31, 33, 35, 66–68]. THB8 to THB12 form a group of algal 2/2Hbs that feature moderately elongated N- and considerably elongated C-termini, extending the HBD by about 50 N-terminal and 225 (THB11) to 536 (THB9) C-terminal amino acids. THB10 and THB11, two members of this group that have been studied in their recombinant holo forms, are pentacoordinate in the reduced state (heme-Fe^II^) [31], whereas the single-domain algal globins THB1, -2 and -4 are hexacoordinate [29, 30, 33]. Although the role of hexa- versus pentacoordination, found in both 2/2Hbs and 3/3Hbs, is still unclear, the different structure–function relationships observed in hexa- and pentacoordinate globins indicate specialized physiological functions [23]. Here, we solved the crystal structure of the THB11 HBD, in order to widen the structural knowledge base for understanding the biochemical, and, ultimately, physiological functions of the versatile 2/2Hbs from *Chlamydomonas*. The tertiary structure of the THB11 HBD recapitulates the overall 2/2Hb topology well (Figs. 1, S3), but it reveals several features that are likely to result in specific reactivities. These are the noticeable kink in helix E, the tilted heme plane, a distal site of intermediate polarity, and an unusual accumulation of Met residues at topological sites known from other class I 2/2Hbs to form tunnel openings.

The tilt of the heme plane appears to be a unique feature of THB11-NC when compared to additional class I 2/2Hbs (Figs. 1b, S3), and might be related to the kink in helix E. Although this particular conformation might be an artificial consequence from the rigid crystal packing, and/or a result of the deletion of the N- and C-termini, the observed heme tilting might contribute to the marked asymmetry of the Fe center we observed by EXAFS analyses of the deoxy (Fe^II^) and oxy forms (Fe^II^-O_2_) of full-length THB11 as well as THB11-C [31]. Overall, the distances between the heme-Fe and surrounding first, second and third sphere atoms measured in the cyanomet THB11-NC crystal structure are comparable to the EXAFS simulation parameters obtained with oxy full-length THB11 and THB11-C (Fig. S6 in the supplementary material), suggesting that neither crystallization nor the removal of the termini resulted in large deviations from the actual protein structure in solution. The kink in the E-helix might be related to the absence of the Gly–Gly motif present in many class I 2/2Hb primary sequences directly following the residues forming helix E [13, 14] (Fig. S4 in the supplementary material). Together with Gly motifs after the A- and preceding the F-helix, these are thought to provide the flexibility required to stabilize the truncated 2/2Hb fold [25]. In the THB11 primary sequence, the E-helix is followed instead by a Gly–Pro–Pro motif, which is likely to result in a strongly decreased flexibility of the EF-turn.

The heme tilt observed in THB11-NC goes along with a leaning of the HisF8 imidazole plane towards the porphyrin, a conformation that is related to a lower O_2_ affinity in hemoglobin α [4], and references therein]. On the other hand, the THB11 HisF8 plane is in a moderately staggered position, which has been associated with a higher O_2_ affinity, especially when the Nδ of HisF8 forms a H-bond to a carbonyl group [69], which is the case in THB11-NC (Fig. 2a, b). Additionally, distortions of protein-bound heme groups from an optimally planar and symmetric system are common [70] and can play major roles in determining the reactivity of the heme–iron, such as ligand affinity and redox potentials [71–73]. With an out of plane displacement of about 0.5 Å, the heme group of THB11-NC features a small heme distortion compared to other class I 2/2Hbs [74]. Its dominant modes, doming and ruffling, are often observed among this class of truncated hemoglobins, although ruffling seems to be slightly more common [74].

While we did not determine ligand/substrate affinities for THB11 and its length variants, we noted that all proteins were consistently purified in their oxy forms. These states were stable throughout the purification process (e.g. see Fig. 4d), suggesting that the O_2_ ligand was well stabilized by all protein variants. This notion is supported by a preliminary determination of autoxidation rates. These vary between 0.004 h^−1^ (THB11-N) and 0.01 h^−1^ (full-length THB11) and were thus comparable to the autoxidation rate of Mb [75]. H-bond networks that stabilize heme-bound ligands and result in low ligand dissociation rates are observed frequently in the distal site of group I 2/2Hbs. TyrB10 is the most conserved [13, 14] and forms a direct H-bond to the heme-bound ligand [6, 53, 76, 77]. In several cases, the ligand also forms H-bonds to GlnE7, such as in *C. eugametos*, *Paramecium caudatum* and *T. pyriformis* 2/2Hbs [6, 55], whereas TyrB10 is additionally stabilized by H-bonds to a polar E7 and/or E11 residue [6, 53, 55]. A polar E7 residue is not essential for a strong ligand stabilization, as shown for *M. tuberculosis* GlbN, which features LeuE7, but a high O_2_ affinity and a low autoxidation rate [76]. In the THB11 HBD crystal structure, the CN^–^ ligand is in a strong H-bond distance only to TyrB10(32), which, in turn, is in moderate H-bond distance to GlnE11(57), similar to the situation in *M. tuberculosis* GlbN [54, 76, 78]. In *M. tuberculosis* cyanomet GlbN, however, the CN^–^ ligand is additionally in weak H-bond distance (3.4 Å) to GlnE11 [53], whereas this distance is at least 4.0 Å in THB11-NC, which might result in a less pronounced stabilization of a given ligand, consistent with autoxidation rates five- to ten times higher than in *M. tuberculosis* GlbN [76].

The reactivity of a given globin is also strongly dependent on access and escape routes through which ligands and substrates/products can enter or escape. In many 2/2Hbs, tunnels and cavities have been observed such as the L-shaped system of a long (LT) and a short tunnel (ST) in the class I 2/2Hbs from *M. tuberculosis*, *T. pyriformis* and *C. eugametos* [21, 54, 55] (also see Fig. 3a). The tunnels and cavities are dynamic and dependent on the presence or absence of ligands. In the crystal structures of the class I hexacoordinate 2/2Hbs from *Synechocystis* and *Synechococcus*, for example, access tunnels are only observed when a ligand is bound to the heme-Fe [52, 74]. In case of *M. tuberculosis* GlbN, which has a high nitric oxide (NO) dioxygenase activity, it is proposed that O_2_ enters the protein through the ST, and that binding of the O_2_ molecule to deoxy-GlbN triggers the opening of the LT through a gating mechanism operated by PheE15 [58, 59, 79, 80]. Thus, the missing ST in THB11-NC has to be interpreted with caution, and ligands might simply enter or leave through the exposed heme edge in any case. However, it is noteworthy that the LT can be computed within the THB11 HBD structure (Fig. 3a), and that the residues that form the LT aperture in *M. tuberculosis* and *T. pyriformis* class I 2/2Hbs are similar in THB11 [21, 54, 55] (Figs. S4, S7 in the supplementary material). In contrast, the clustering of Met residues at the possible ST aperture as observed in THB11 appears to be unusual. These positions (PheG5, AlaG9, LeuH8, IleH11 and AlaH12 in *M. tuberculosis* GlbN) are mostly aliphatic in other class I 2/2Hbs, whereas several of the algal Hbs, including THB1, possess Met residues at one or the other position [13] (Figs. S4, S7). The H11 site has been implicated to be of importance for ligand access by computational approaches [14]. IleH11 is indeed shifted in the isobutyl cyanide derivative of *M. tuberculosis* GlbN, and both IleH11 and AlaG9 are shifted in the xenon adduct, causing the ST entrance to widen [21]. In the THB11-NC structure, MetH11(123) as well as the adjacent LeuH12(124) were modeled in two conformations, and, together with MetG9(99), MetH11(123) restricts the site at which the ST exits in *M. tuberculosis* GlbN (Fig. 3). In silico mutagenesis of both residues (M99A and M123I) resulted in a model in which an ST very similar to that of *M. tuberculosis* GlbN was computed by Caver (Fig. S8 in the supplementary material).

It is tempting to speculate that the Met clustering could play a particular role for the regulation of ligand access in THB11, especially in the context of an algal host that photosynthetically produces reactive oxygen species on a regular basis. Met residues are prone to oxidation, a process that is reversible by methionine sulfoxide reductases and employed for antioxidative defense as well as for protein activity regulation [81]. We do not know where the THB11 C terminus would be located and if it would shield the ST access site, respectively. Also, most of the Met residues found at the putative ST aperture in THB11-NC are buried within the protein. However, depending on the surrounding flexibility, even buried Met side chains can be oxidized [82], and both MetG9(99) and MetH8(120) are close to the surface.

Structurally, Met residues are often involved in hydrophobic interactions. From the THB11 HBD crystal structure, we cannot infer the positions of the N- and C-termini relative to the HBD. However, it is noteworthy that the C terminus consists of hydrophobic amino acids to almost 70%. It is very Ala-rich (about 36%) and contains two Phe, one Trp and one Tyr. The latter three residues are often involved in stabilizing Met-aromatic motifs [83]. It is possible that these amino acids confer interactions of the termini (the N terminus also contains one Phe and one Trp each) to the HBD, in whose structure, as noted above, the putative ST aperture residues MetG9(99) and MetH8(120) are close to, and Met51 and Met59 are directly at the surface. Met-aromatic interactions might also confer the intermolecular interactions we infer from analyses of the oligomeric states of the THB11 length variants containing at least one of the termini (Fig. 4), but these might also or additionally be promoted by other hydrophobic interactions or by disulfide bridges, as both termini contain one Cys each.

A physiological relevance of the THB11 differential oligomerization remains to be proven, especially as the in vivo role of THB11 has not been investigated yet. THB11 is not predicted by the algae-specific localization prediction tool PredAlgo [84] to be localized in an organelle, whereas peptides of THB11 have been detected in the mitochondria-enriched fraction [85]. *THB11* transcript levels are higher in *Chlamydomonas* cells in the dark [31, 86] and increase steadily in the subjective night in synchronized algal cultures [66, 87], when also THB11 peptides can be detected [87]. These data might suggest a differential function of THB11 in the night versus the day, and the different intracellular metabolic states present under these conditions might well influence the oligomerization status of the protein. This, in turn, might influence reaction kinetics, as the absence of the C-terminus results in a reduction of the nitrite reduction rate by about 50% (Fig. 5). Although our current data do not allow us to distinguish between a role of the C-terminus by itself or by its influence on the oligomerization status, extensions of the globin domain have been shown before to influence the reactivities of globins, such as the pre-A motif of *M. tuberculosis* GlbN, which is crucial for the high NO dioxygenase activity of the protein. It has been proposed that its absence influences protein dynamics in a way that the PheE15 gate constitutively blocks the long tunnel [88], whereas more recent results suggest that the pre-A motif prevents the formation of stable GlbN dimers, in which the short tunnel is blocked, and the long tunnel restricted [89]. In *Methanosarcina acetivorans* protoglobin, a 3/3Hb, dimerization has instead been calculated to result in an opening of the tunnels [90]. The single class II 2/2Hb from *A. thaliana* has comparably short N- (25 residues) and C-terminal (32 residues) extensions, however, these appear to modulate its oligomeric state (both termini), the coordination of its heme-Fe (N-terminus) as well as azide binding to its ferric state (C-terminus) [91].

To date, although many different Hb types have been shown to catalyze nitrite reduction to NO, generalizable properties that are rate-determining are not known. Factors that have been discussed are hexa- *versus* pentacoordination, H-bond donors in the distal site, distal site volume, the redox potential, and hydrophobic cavities and tunnels [60, 64, 92, 93]. Noteworthy, the importance of a given structural or biochemical element appears to vary in different globin types. For example, pentacoordinate variants of the naturally hexacoordinate 3/3Hb neuroglobin exhibit strongly increased activities [64, 93], whereas a similar mutation in *Arabidopsis* class I nonsymbiotic Hb has hardly any effect [92]. In general, as has also been discussed by Sturms et al. [61], hexa- or pentacoordination is not suitable to predict nitrite conversion velocity, since the groups of penta- and hexacoordinate Hbs both contain high- and low-efficiency nitrite reductases.

The nitrite reduction rates of *Chlamydomonas* THB11 and its variants were higher than those of most animal Hbs studied at similar pH values, for which rates from 0.062 M^−1^ s^−1^ (human neuroglobin with reduced cysteine residues; Tiso et al. [64]) to 6 M^−1^ s^−1^ (human RBC-Hb in the R-state; Huang et al. [94]) have been reported [see a compilation of rates in [60]. THB11 is thus more comparable to algal, plant and bacterial Hbs with reported nitrite reduction rates of ca. 46 M^−1^ s^−1^ (this study) or 98 M^−1^ s^−1^ [30] for *Chlamydomonas* THB1, 54 M^−1^ s^−1^ for THB2 [30], 19 M^−1^ s^−1^ and 83 M^−1^ s^−1^ for *Arabidopsis* and rice class I nonsymbiotic Hbs (3/3Hbs), respectively, or 68 M^−1^ s^−1^ for *Synechocystis* GlbN [61, 62]. As explained above, it is hardly possible to ascribe the comparably high nitrite reduction rate to any given feature of THB11, although the putative H-bond stabilizing network in the distal site and the observed (LT, heme edge) or suggested (ST) access routes might contribute to efficient ligand stabilization and access or escape. Likewise, we do not know whether nitrite reduction by globins plays a physiological role in *Chlamydomonas*, and if so, which. It has been suggested that the comparably high nitrite reduction activities observed for several Hbs from photosynthetic organisms might be employed for nitrite detoxification and/or for NO generation [61, 62]. Within the cellular environment, it is relevant that Hb-catalyzed nitrite reduction requires the ferric heme to be re-reduced to its ferrous form. It may be in this context that the ferric hexacoordination of THB11 indicated by UV–Vis spectroscopy plays a role, because the presence of a sixth ligand can influence both the redox potential and reduction kinetics of a globin [95]. In any case, an adequate reductase or reducing equivalent must be present. *Chlamydomonas* THB1, -2 and -4 can be reduced by both the diaphorase domain of nitrate reductase and by a ferredoxin – ferredoxin:nicotinamide adenine dinucleotide phosphate reductase system [29, 35], but this has not been tested for the additional algal Hbs. Still, nitrite reduction to NO by (one of) the algal 2/2Hbs might be one route of NO production in *Chlamydomonas*. Whereas nitrate reductase has been shown to be involved in NO generation in the alga [96, 97], many laboratory strains are deficient for this enzyme. These strains still appear to employ NO-based signaling pathways [27, 98–100], and nitrite as a source for NO production has been suggested [99, 100]. Because NO usually binds to deoxyglobins with a high affinity, a role of globins in free NO generation for signaling purposes is still controversial. However, free NO accumulates upon nitrite reduction by *Arabidopsis* Hbs [62]. Also, it has been shown that deoxy-Mb, in the presence of nitrite, can inhibit mitochondrial respiration [101], and NO production from nitrite by cytoglobin was correlated with a higher activity of NO-sensitive guanylate cyclase [102].

In summary, we have solved the structure of the HBD of one of the ferrous pentacoordinate *Chlamydomonas* 2/2Hbs, THB11, demonstrating several unique features that are likely to result in a specific function of the protein. The cellular role of the extended termini remains to be investigated, but our studies on THB11 length variants show that they prompt oligomerization in vitro, suggesting that they confer protein–protein interactions also in the cell. The effect of the absence of the C terminus on nitrite reduction rates indicates that it can regulate THB11 activity, which is the first hint for a function of the algae-specific extensions of a conserved 2/2Hb core.

## Electronic supplementary material

Below is the link to the electronic supplementary material.

Supplementary file1 (PDF 2037 kb)

## Abbreviations

AEC: Anion exchange chromatography
EXAFS: Extended X-ray absorption fine structure
FHb: Flavohemoglobin
GCS: Globin-coupled sensor
Hb: Hemoglobin
2/2Hb: Hemoglobin with a 2-over-2 helical fold
3/3Hb: Hemoglobin with a 3-over-3 helical fold
HBD: Heme-binding domain
Mb: Myoglobin
NaDt: Sodium dithionite
RBC-Hb: Red blood cell hemoglobin
SEC: Size exclusion chromatography
THB: Truncated hemoglobin

## References

[1] Vinogradov SN, Hoogewijs D, Bailly X, Mizuguchi K, Dewilde S, Moens L, Vanfleteren JR (2007). A model of globin evolution. Gene.

[2] Vinogradov SN, Hoogewijs D, Bailly X, Arredondo-Peter R, Guertin M, Gough J, Dewilde S, Moens L, Vanfleteren JR (2005). Three globin lineages belonging to two structural classes in genomes from the three kingdoms of life. Proc Natl Acad Sci USA.

[3] Vinogradov SN, Moens L (2008). Diversity of globin function: enzymatic, transport, storage, and sensing. J Biol Chem.

[4] Gell DA (2018). Structure and function of haemoglobins. Blood Cells Mol Dis.

[5] Perutz MF, Kendrew JC, Watson HC (1965). Structure and function of haemoglobin: II. Some relations between polypeptide chain configuration and amino acid sequence. J Mol Biol.

[6] Pesce A, Couture M, Dewilde S, Guertin M, Yamauchi K, Ascenzi P, Moens L, Bolognesi M (2000). A novel two-over-two alpha-helical sandwich fold is characteristic of the truncated hemoglobin family. EMBO J.

[7] Bonamore A, Boffi A (2008). Flavohemoglobin: structure and reactivity. IUBMB Life.

[8] Martínková M, Kitanishi K, Shimizu T (2013). Heme-based globin-coupled oxygen sensors: linking oxygen binding to functional regulation of diguanylate cyclase, histidine kinase, and methyl-accepting chemotaxis. J Biol Chem.

[9] Vinogradov SN, Bailly X, Smith DR, Tinajero-Trejo M, Poole RK, Hoogewijs D (2013). Microbial eukaryote globins. Adv Microb Physiol.

[10] Vinogradov SN, Tinajero-Trejo M, Poole RK, Hoogewijs D (2013). Bacterial and archaeal globins—a revised perspective. Biochim Biophys Acta.

[11] Vázquez-Limón C, Hoogewijs D, Vinogradov SN, Arredondo-Peter R (2012). The evolution of land plant hemoglobins. Plant Sci.

[12] Wittenberg JB, Bolognesi M, Wittenberg BA, Guertin M (2002). Truncated hemoglobins: a new family of hemoglobins widely distributed in bacteria, unicellular eukaryotes, and plants. J Biol Chem.

[13] Vuletich DA, Lecomte JT (2006). A phylogenetic and structural analysis of truncated hemoglobins. J Mol Evol.

[14] Bustamante JP, Radusky L, Boechi L, Estrin DA, Ten Have A, Marti MA (2016). Evolutionary and functional relationships in the truncated hemoglobin family. PLoS Comput Biol.

[15] Vinogradov SN, Hoogewijs D, Bailly X, Arredondo-Peter R, Gough J, Dewilde S, Moens L, Vanfleteren JR (2006). A phylogenomic profile of globins. BMC Evol Biol.

[16] Vinogradov SN, Fernández I, Hoogewijs D, Arredondo-Peter R (2011). Phylogenetic relationships of 3/3 and 2/2 hemoglobins in Archaeplastida genomes to bacterial and other eukaryote hemoglobins. Mol Plant.

[17] Hade MD, Kaur J, Chakraborti PK, Dikshit KL (2017). Multidomain truncated hemoglobins: new members of the globin family exhibiting tandem repeats of globin units and domain fusion. IUBMB Life.

[18] Stewart JJ, Coyne KJ (2011). Analysis of raphidophyte assimilatory nitrate reductase reveals unique domain architecture incorporating a 2/2 hemoglobin. Plant Mol Biol.

[19] Bonamore A, Attili A, Arenghi F, Catacchio B, Chiancone E, Morea V, Boffi A (2007). A novel chimera: the "truncated hemoglobin-antibiotic monooxygenase" from *Streptomyces avermitilis*. Gene.

[20] Estarellas C, Capece L, Seira C, Bidon-Chanal A, Estrin DA, Luque FJ (2016). Structural plasticity in globins: role of protein dynamics in defining ligand migration pathways. Adv Protein Chem Struct Biol.

[21] Milani M, Pesce A, Ouellet Y, Dewilde S, Friedman J, Ascenzi P, Guertin M, Bolognesi M (2004). Heme-ligand tunneling in group I truncated hemoglobins. J Biol Chem.

[22] Goldbeck RA, Bhaskaran S, Ortega C, Mendoza JL, Olson JS, Soman J, Kliger DS, Esquerra RM (2006). Water and ligand entry in myoglobin: assessing the speed and extent of heme pocket hydration after CO photodissociation. Proc Natl Acad Sci USA.

[23] Kakar S, Hoffman FG, Storz JF, Fabian M, Hargrove MS (2010). Structure and reactivity of hexacoordinate hemoglobins. Biophys Chem.

[24] Capece L, Boechi L, Perissinotti LL, Arroyo-Mañez P, Bikiel DE, Smulevich G, Marti MA, Estrin DA (2013). Small ligand–globin interactions: reviewing lessons derived from computer simulation. Biochim Biophys Acta Prot Proteom.

[25] Pesce A, Bolognesi M, Nardini M (2013). The diversity of 2/2 (truncated) globins. Adv Microb Physiol.

[26] Smagghe BJ, Hoy JA, Percifield R, Kundu S, Hargrove MS, Sarath G, Hilbert JL, Watts RA, Dennis ES, Peacock WJ, Dewilde S, Moens L, Blouin GC, Olson JS, Appleby CA (2009). Review: correlations between oxygen affinity and sequence classifications of plant hemoglobins. Biopolymers.

[27] Hemschemeier A, Düner M, Casero D, Merchant SS, Winkler M, Happe T (2013). Hypoxic survival requires a 2-on-2 hemoglobin in a process involving nitric oxide. Proc Natl Acad Sci USA.

[28] Johnson EA, Lecomte JTJ, Robert KP (2015). The Haemoglobins of Algae. Advances in microbial physiology.

[29] Johnson EA, Russo MM, Nye DB, Schlessman JL, Lecomte JTJ (2018). Lysine as a heme iron ligand: a property common to three truncated hemoglobins from *Chlamydomonas reinhardtii*. Biochim Biophys Acta Gen Subj.

[30] Ciaccio C, Ocaña-Calahorro F, Droghetti E, Tundo GR, Sanz-Luque E, Polticelli F, Visca P, Smulevich G, Ascenzi P, Coletta M (2015). Functional and spectroscopic characterization of *Chlamydomonas reinhardtii* truncated hemoglobins. PLoS ONE.

[31] Huwald D, Schrapers P, Kositzki R, Haumann M, Hemschemeier A (2015). Characterization of unusual truncated hemoglobins of *Chlamydomonas reinhardtii* suggests specialized functions. Planta.

[32] Rice SL, Boucher LE, Schlessman JL, Preimesberger MR, Bosch J, Lecomte JT (2015). Structure of *Chlamydomonas reinhardtii* THB1, a group 1 truncated hemoglobin with a rare histidine-lysine heme ligation. Acta Crystallogr F Struct Biol Commun.

[33] Johnson EA, Rice SL, Preimesberger MR, Nye DB, Gilevicius L, Wenke BB, Brown JM, Witman GB, Lecomte JT (2014). Characterization of THB1, a *Chlamydomonas reinhardtii* truncated hemoglobin: linkage to nitrogen metabolism and identification of lysine as the distal heme ligand. Biochemistry (Mosc).

[34] Johnson EA, Lecomte JT (2014). Characterization of the truncated hemoglobin THB1 from protein extracts of Chlamydomonas reinhardtii. F1000Res.

[35] Sanz-Luque E, Ocaña-Calahorro F, de Montaigu A, Chamizo-Ampudia A, Llamas A, Galván A, Fernández E (2015). THB1, a truncated hemoglobin, modulates nitric oxide levels and nitrate reductase activity. Plant J.

[36] Nye DB, Johnson EA, Mai MH, Lecomte JTJ (2019). Replacement of the heme axial lysine as a test of conformational adaptability in the truncated hemoglobin THB1. J Inorg Biochem.

[37] Laemmli UK (1970). Cleavage of structural proteins during the assembly of the head of bacteriophage T4. Nature.

[38] Tropea JE, Cherry S, Waugh DS (2009). Expression and purification of soluble His_6_-tagged TEV protease. Methods Mol Biol.

[39] Simpson RJ (2007). Zinc/Imidazole procedure for visualization of proteins in gels by negative staining. CSH Protoc.

[40] Waltersperger S, Olieric V, Pradervand C, Glettig W, Salathe M, Fuchs MR, Curtin A, Wang X, Ebner S, Panepucci E, Weinert T, Schulze-Briese C, Wang M (2015). PRIGo: a new multi-axis goniometer for macromolecular crystallography. J Synchrotron Radiat.

[41] Kabsch W (2010). XDS. Acta Crystallogr D Biol Crystallogr.

[42] Sheldrick GM (2008). A short history of SHELX. Acta Crystallogr A.

[43] Pape T, Schneider TR (2004). *HKL2MAP*: a graphical user interface for macromolecular phasing with *SHELX* programs. J Appl Cryst.

[44] Adams PD, Afonine PV, Bunkóczi G, Chen VB, Davis IW, Echols N, Headd JJ, Hung LW, Kapral GJ, Grosse-Kunstleve RW, McCoy AJ, Moriarty NW, Oeffner R, Read RJ, Richardson DC, Richardson JS, Terwilliger TC, Zwart PH (2010). *PHENIX*: a comprehensive Python-based system for macromolecular structure solution. Acta Crystallogr D Biol Crystallogr.

[45] Liebschner D, Afonine PV, Baker ML, Bunkóczi G, Chen VB, Croll TI, Hintze B, Hung LW, Jain S, McCoy AJ, Moriarty NW, Oeffner RD, Poon BK, Prisant MG, Read RJ, Richardson JS, Richardson DC, Sammito MD, Sobolev OV, Stockwell DH, Terwilliger TC, Urzhumtsev AG, Videau LL, Williams CJ, Adams PD (2019). Macromolecular structure determination using X-rays, neutrons and electrons: recent developments in *Phenix*. Acta Crystallogr D Struct Biol.

[46] Emsley P, Cowtan K (2004). *Coot*: model-building tools for molecular graphics. Acta Crystallogr D Biol Crystallogr.

[47] Pavelka A, Sebestova E, Kozlikova B, Brezovsky J, Sochor J, Damborsky J (2016). CAVER: algorithms for analyzing dynamics of tunnels in macromolecules. IEEE/ACM Trans Comput Biol Bioinform.

[48] Jentzen W, Ma JG, Shelnutt JA (1998). Conservation of the conformation of the porphyrin macrocycle in hemoproteins. Biophys J.

[49] Jentzen W, Song X-Z, Shelnutt JA (1997). Structural characterization of synthetic and protein-bound porphyrins in terms of the lowest-frequency normal coordinates of the macrocycle. J Phys Chem B.

[50] Holm L, Laakso LM (2016). Dali server update. Nucleic Acids Res.

[51] Sievers F, Wilm A, Dineen D, Gibson TJ, Karplus K, Li W, Lopez R, McWilliam H, Remmert M, Söding J, Thompson JD, Higgins DG (2011). Fast, scalable generation of high-quality protein multiple sequence alignments using Clustal Omega. Mol Syst Biol.

[52] Trent JT, Kundu S, Hoy JA, Hargrove MS (2004). Crystallographic analysis of *Synechocystis* cyanoglobin reveals the structural changes accompanying ligand binding in a hexacoordinate hemoglobin. J Mol Biol.

[53] Milani M, Ouellet Y, Ouellet H, Guertin M, Boffi A, Antonini G, Bocedi A, Mattu M, Bolognesi M, Ascenzi P (2004). Cyanide binding to truncated hemoglobins: a crystallographic and kinetic study. Biochemistry (Mosc).

[54] Milani M, Pesce A, Ouellet Y, Ascenzi P, Guertin M, Bolognesi M (2001). *Mycobacterium tuberculosis* hemoglobin N displays a protein tunnel suited for O_2_ diffusion to the heme. EMBO J.

[55] Igarashi J, Kobayashi K, Matsuoka A (2011). A hydrogen-bonding network formed by the B10–E7–E11 residues of a truncated hemoglobin from *Tetrahymena pyriformis* is critical for stability of bound oxygen and nitric oxide detoxification. J Biol Inorg Chem.

[56] Scott NL, Xu Y, Shen G, Vuletich DA, Falzone CJ, Li Z, Ludwig M, Pond MP, Preimesberger MR, Bryant DA, Lecomte JT (2010). Functional and structural characterization of the 2/2 hemoglobin from *Synechococcus* sp. PCC 7002. Biochemistry (Mosc).

[57] Falzone CJ, Christie VuB, Scott NL, Lecomte JT (2002). The solution structure of the recombinant hemoglobin from the cyanobacterium *Synechocystis* sp. PCC 6803 in its hemichrome state. J Mol Biol.

[58] Oliveira A, Singh S, Bidon-Chanal A, Forti F, Martí MA, Boechi L, Estrin DA, Dikshit KL, Luque FJ (2012). Role of PheE15 gate in ligand entry and nitric oxide detoxification function of *Mycobacterium tuberculosis* truncated hemoglobin N. PLoS ONE.

[59] Bidon-Chanal A, Martí MA, Estrin DA, Luque FJ (2007). Dynamical regulation of ligand migration by a gate-opening molecular switch in truncated hemoglobin-N from *Mycobacterium tuberculosis*. J Am Chem Soc.

[60] Tejero J, Gladwin MT (2014). The globin superfamily: functions in nitric oxide formation and decay. Biol Chem.

[61] Sturms R, DiSpirito AA, Hargrove MS (2011). Plant and cyanobacterial hemoglobins reduce nitrite to nitric oxide under anoxic conditions. Biochemistry (Mosc).

[62] Tiso M, Tejero J, Kenney C, Frizzell S, Gladwin MT (2012). Nitrite reductase activity of nonsymbiotic hemoglobins from *Arabidopsis thaliana*. Biochemistry (Mosc).

[63] Brooks J (1937). The action of nitrite on haemoglobin in the absence of oxygen. Proc R Soc Lond B Biol Sci.

[64] Tiso M, Tejero J, Basu S, Azarov I, Wang X, Simplaceanu V, Frizzell S, Jayaraman T, Geary L, Shapiro C, Ho C, Shiva S, Kim-Shapiro DB, Gladwin MT (2011). Human neuroglobin functions as a redox-regulated nitrite reductase. J Biol Chem.

[65] Yi J, Heinecke J, Tan H, Ford PC, Richter-Addo GB (2009). The distal pocket histidine residue in horse heart myoglobin directs the *O*-binding mode of nitrite to the heme iron. J Am Chem Soc.

[66] Zones JM, Blaby IK, Merchant SS, Umen JG (2015). High-resolution profiling of a synchronized diurnal transcriptome from *Chlamydomonas reinhardtii* reveals continuous cell and metabolic differentiation. Plant Cell.

[67] Hemschemeier A, Casero D, Liu B, Benning C, Pellegrini M, Happe T, Merchant SS (2013). COPPER RESPONSE REGULATOR1-dependent and -independent responses of the *Chlamydomonas reinhardtii* transcriptome to Dark Anoxia. Plant Cell.

[68] Minaeva E, Zalutskaya Z, Filina V, Ermilova E (2017). Truncated hemoglobin 1 is a new player in *Chlamydomonas reinhardtii* acclimation to sulfur deprivation. PLoS ONE.

[69] Capece L, Marti MA, Crespo A, Doctorovich F, Estrin DA (2006). Heme protein oxygen affinity regulation exerted by proximal effects. J Am Chem Soc.

[70] Shelnutt JA, Song X-Z, Ma J-G, Jia S-L, Jentzen W, Medforth JC (1998). Nonplanar porphyrins and their significance in proteins. Chem Soc Rev.

[71] Imada Y, Nakamura H, Takano Y (2018). Density functional study of porphyrin distortion effects on redox potential of heme. J Comput Chem.

[72] Bikiel DE, Forti F, Boechi L, Nardini M, Luque FJ, Marti MA, Estrin DA (2010). Role of heme distortion on oxygen affinity in heme proteins: the protoglobin case. J Phys Chem B.

[73] Van Doorslaer S, Tilleman L, Verrept B, Desmet F, Maurelli S, Trandafir F, Moens L, Dewilde S (2012). Marked difference in the electronic structure of cyanide-ligated ferric protoglobins and myoglobin due to heme ruffling. Inorg Chem.

[74] Wenke BB, Lecomte JT, Héroux A, Schlessman JL (2014). The 2/2 hemoglobin from the cyanobacterium *Synechococcus* sp. PCC 7002 with covalently attached heme: comparison of X-ray and NMR structures. Proteins.

[75] Brantley RE, Smerdon SJ, Wilkinson AJ, Singleton EW, Olson JS (1993). The mechanism of autooxidation of myoglobin. J Biol Chem.

[76] Couture M, Yeh SR, Wittenberg BA, Wittenberg JB, Ouellet Y, Rousseau DL, Guertin M (1999). A cooperative oxygen-binding hemoglobin from *Mycobacterium tuberculosis*. Proc Natl Acad Sci USA.

[77] Couture M, Das TK, Lee HC, Peisach J, Rousseau DL, Wittenberg BA, Wittenberg JB, Guertin M (1999). *Chlamydomonas* chloroplast ferrous hemoglobin. Heme pocket structure and reactions with ligands. J Biol Chem.

[78] Yeh SR, Couture M, Ouellet Y, Guertin M, Rousseau DL (2000). A cooperative oxygen binding hemoglobin from *Mycobacterium tuberculosis*. Stabilization of heme ligands by a distal tyrosine residue. J Biol Chem.

[79] Daigle R, Rousseau JA, Guertin M, Lagüe P (2009). Theoretical investigations of nitric oxide channeling in *Mycobacterium tuberculosis* truncated hemoglobin N. Biophys J.

[80] Crespo A, Martí MA, Kalko SG, Morreale A, Orozco M, Gelpi JL, Luque FJ, Estrin DA (2005). Theoretical study of the truncated hemoglobin HbN: exploring the molecular basis of the NO detoxification mechanism. J Am Chem Soc.

[81] Lim JM, Kim G, Levine RL (2018). Methionine in proteins: it's not just for protein initiation anymore. Neurochem Res.

[82] Xu K, Uversky VN, Xue B (2012). Local flexibility facilitates oxidization of buried methionine residues. Protein Pept Lett.

[83] Valley CC, Cembran A, Perlmutter JD, Lewis AK, Labello NP, Gao J, Sachs JN (2012). The methionine–aromatic motif plays a unique role in stabilizing protein structure. J Biol Chem.

[84] Tardif M, Atteia A, Specht M, Cogne G, Rolland N, Brugière S, Hippler M, Ferro M, Bruley C, Peltier G, Vallon O, Cournac L (2012). PredAlgo: a new subcellular localization prediction tool dedicated to green algae. Mol Biol Evol.

[85] Terashima M, Specht M, Naumann B, Hippler M (2010). Characterizing the anaerobic response of *Chlamydomonas reinhardtii* by quantitative proteomics. Mol Cell Proteom.

[86] Duanmu D, Casero D, Dent RM, Gallaher S, Yang W, Rockwell NC, Martin SS, Pellegrini M, Niyogi KK, Merchant SS, Grossman AR, Lagarias JC (2013). Retrograde bilin signaling enables *Chlamydomonas* greening and phototrophic survival. Proc Natl Acad Sci USA.

[87] Strenkert D, Schmollinger S, Gallaher SD, Salomé PA, Purvine SO, Nicora CD, Mettler-Altmann T, Soubeyrand E, Weber APM, Lipton MS, Basset GJ, Merchant SS (2019). Multiomics resolution of molecular events during a day in the life of Chlamydomonas. Proc Natl Acad Sci USA.

[88] Lama A, Pawaria S, Bidon-Chanal A, Anand A, Gelpí JL, Arya S, Martí M, Estrin DA, Luque FJ, Dikshit KL (2009). Role of Pre-A motif in nitric oxide scavenging by truncated hemoglobin, HbN, of *Mycobacterium tuberculosis*. J Biol Chem.

[89] Pesce A, Bustamante JP, Bidon-Chanal A, Boechi L, Estrin DA, Luque FJ, Sebilo A, Guertin M, Bolognesi M, Ascenzi P, Nardini M (2016). The N-terminal pre-A region of *Mycobacterium tuberculosis* 2/2HbN promotes NO-dioxygenase activity. FEBS J.

[90] Forti F, Boechi L, Bikiel D, Martí MA, Nardini M, Bolognesi M, Viappiani C, Estrin D, Luque FJ (2011). Ligand migration in *Methanosarcina acetivorans* protoglobin: effects of ligand binding and dimeric assembly. J Phys Chem B.

[91] Mukhi N, Dhindwal S, Uppal S, Kapoor A, Arya R, Kumar P, Kaur J, Kundu S (2016). Structural and functional significance of the N- and C-terminal appendages in *Arabidopsis* truncated hemoglobin. Biochemistry (Mosc).

[92] Kumar N, Astegno A, Chen J, Giorgetti A, Dominici P (2016). Residues in the Distal Heme Pocket of Arabidopsis non-symbiotic hemoglobins: implication for nitrite reductase activity. Int J Mol Sci.

[93] Tejero J, Sparacino-Watkins CE, Ragireddy V, Frizzell S, Gladwin MT (2015). Exploring the mechanisms of the reductase activity of neuroglobin by site-directed mutagenesis of the heme distal pocket. Biochemistry (Mosc).

[94] Huang Z, Shiva S, Kim-Shapiro DB, Patel RP, Ringwood LA, Irby CE, Huang KT, Ho C, Hogg N, Schechter AN, Gladwin MT (2005). Enzymatic function of hemoglobin as a nitrite reductase that produces NO under allosteric control. J Clin Invest.

[95] Weiland TR, Kundu S, Trent JT, Hoy JA, Hargrove MS (2004). Bis-histidyl hexacoordination in hemoglobins facilitates heme reduction kinetics. J Am Chem Soc.

[96] Sakihama Y, Nakamura S, Yamasaki H (2002). Nitric oxide production mediated by nitrate reductase in the green alga *Chlamydomonas reinhardtii*: an alternative NO production pathway in photosynthetic organisms. Plant Cell Physiol.

[97] Chamizo-Ampudia A, Sanz-Luque E, Llamas A, Ocaña-Calahorro F, Mariscal V, Carreras A, Barroso JB, Galván A, Fernández E (2016). A dual system formed by the ARC and NR molybdoenzymes mediates nitrite-dependent NO production in *Chlamydomonas*. Plant Cell Environ.

[98] Düner M, Lambertz J, Mügge C, Hemschemeier A (2018). The soluble guanylate cyclase CYG12 is required for the acclimation to hypoxia and trophic regimes in *Chlamydomonas reinhardtii*. Plant J.

[99] Wei L, Derrien B, Gautier A, Houille-Vernes L, Boulouis A, Saint-Marcoux D, Malnoë A, Rappaport F, de Vitry C, Vallon O, Choquet Y, Wollman FA (2014). Nitric oxide-triggered remodeling of chloroplast bioenergetics and thylakoid proteins upon nitrogen starvation in *Chlamydomonas reinhardtii*. Plant Cell.

[100] de Mia M, Lemaire SD, Choquet Y, Wollman FA (2018). Nitric oxide remodels the photosynthetic apparatus upon S-starvation in *Chlamydomonas reinhardtii*. Plant Physiol.

[101] Shiva S, Huang Z, Grubina R, Sun J, Ringwood LA, MacArthur PH, Xu X, Murphy E, Darley-Usmar VM, Gladwin MT (2007). Deoxymyoglobin is a nitrite reductase that generates nitric oxide and regulates mitochondrial respiration. Circ Res.

[102] Li H, Hemann C, Abdelghany TM, El-Mahdy MA, Zweier JL (2012). Characterization of the mechanism and magnitude of cytoglobin-mediated nitrite reduction and nitric oxide generation under anaerobic conditions. J Biol Chem.

