# Supplementary material for: Distinctive structural properties of THB11, a pentacoordinate Chlamydomonas reinhardtii truncated hemoglobin with N- and C-terminal extensions
Source: J Biol Inorg Chem. 2020 Feb 11;25(2):267–83. doi: 10.1007/s00775-020-01759-2 (PMC7082302; doi:10.1007/s00775-020-01759-2)
Supplement: Supplementary file 1 — Supplementary file1 (PDF 2037 kb) [file 775_2020_1759_MOESM1_ESM.pdf]

Supplementary material

for

Distinctive structural properties of THB11, a pentacoordinate *Chlamydomonas reinhardtii* truncated hemoglobin with N- and C-terminal extensions

Dennis Huwald[1,2], Sabrina Duda[1], Raphael Gasper[3,4], Vincent Olieric[5], Eckhard Hofmann[3]*, Anja Hemschemeier[1]*

[1]Ruhr University Bochum, Faculty of Biology and Biotechnology, Photobiotechnology, Universitätsstr. 150, 44801 Bochum, Germany

[2]Current address: Charles River Laboratories, Cologne, Germany

[3]Ruhr University Bochum, Faculty of Biology and Biotechnology, Protein Crystallography, Universitätsstr. 150, 44801 Bochum, Germany

[4]Current address: Max-Planck-Institute of Molecular Physiology, Otto-Hahn-Str. 11, 44227 Dortmund, Germany

[5]Swiss Light Source (SLS), Paul-Scherrer-Institute (PSI), 5232 Villigen, Switzerland

***Corresponding authors**

Anja Hemschemeier, e-mail: anja.hemschemeier@rub.de; Eckhard Hofmann, e-mail: eckhard.hofmann@rub.de

ORCID iDs:

Raphael Gasper ORCID iD: 0000-0002-7780-0773; Vincent Olieric ORCID iD: 0000-0002-0533-7222; Eckhard Hofmann ORCID iD: 0000-0003-4874-372X; Anja Hemschemeier ORCID iD: 0000-0001-8879-3348

Table S1

Tab. S1 Overview on constructs and resulting recombinant *C. reinhardtii* 2/2Hb proteins used in this study. The table shows the name of the protein[a], the transcript name on Phytozome 12, *Chlamydomonas reinhardtii v5.5*, the length of the annotated protein and the boundaries of its globin domain[b], and the expression vectors used for recombinant protein synthesis in *E. coli*. Additionally, the residues of the native proteins (as annotated on Phytozome) that are represented by the recombinant proteins and additional features of the latter are indicated. Constructs THB1, THB11 and THB11-C were generated before [1]. -N/-C: deletion of the N-/C-terminus; -NC: deletion of both N- and C-termini.

Name[a]	Transcript name	Protein length (globin domain[b])	Expression vector	Residues of full-length protein	Additional features
**THB1[c]**	*Cre14.g615400.t1.2*	136 (14-127)	pASK-IBA3plus	1-136	C-terminal *Strep*-tag II[d]
**THB11[c]**	*Cre16.g662750.t1.2*	395 (54-169)	pASK-IBA3plus	1-395	C-terminal *Strep*-tag II[d]
**THB11-N**	*Cre16.g662750.t1.2*	395 (54-169)	pASK-IBA5plus	42-395	N-terminal *Strep*-tag II[e] followed by TEV site[f]
**THB11-C[c]**	*Cre16.g662750.t1.2*	395 (54-169)	pASK-IBA3plus	1-180	C-terminal *Strep*-tag II[d]
**THB11-NC**	*Cre16.g662750.t1.2*	395 (54-169)	pASK-IBA5plus	42-180	N-terminal *Strep*-tag II[e] followed by TEV site[f]

[a]Only THB1-THB4 have aliases on Phytozome 12, *Chlamydomonas reinhardtii v5.5*; the names of the additional algal proteins (THB5-THB12) are according to Hemschemeier et al. [2].
[b]The coordinates of the globin domains were updated by analyzing the primary sequences using the National Center for Biotechnology Information's (NCBI's) Protein Basic Local Alignment Search Tool DELTA-BLAST. The CD model cd00454 (TrHb1_N) globin domain coordinates were retrieved from the Conserved Domain Database (CDD).
[c]These constructs were reported before [1].

[d]The C-terminal *Strep*-tag II sequence is preceded by an SA linker (SAWSHPQFEK)
[e]The N-terminal *Strep*-tag II sequences is preceded by an AS- and followed by a GA-linker (ASWSHPQFEKGA)
[f]The TEV recognition site ENLYFQG lies C-terminal of the *Strep*-tag II sequence, preceded by a linker (SGGGENLYFQG)

Table S2

Tab. S2 Oligonucleotides employed to generate recombinant *Chlamydomonas* 2/2Hbs, heterologous production parameters, and resulting protein sequences. Oligonucleotides are listed that were employed to amplify the sequences encoding the indicated proteins for subsequent cloning into expression vectors. Each protein header also indicates the temperature at which heterologous expression was conducted, the OD_{600} of the *E. coli* culture upon induction with anhydrotetracycline, and the duration of the expression. C- and N-terminal *Strep*-tag II sequences of the resulting polypeptide sequences are underlined. N-terminal *Strep*-tag II sequences are followed by a TEV recognition site, written in italic and underlined letters. The TEV cleavage site is indicated by a downward arrow, and the first amino acid of the native protein sequence is separated by a dash sign and written bold type. The residues of THB11-NC which were not resolved in the crystal structure are written in small letters.

**Protein**	**THB1** (*Cre14.g615400.t1.2*)[a] **37°C, OD_{600} 0.6, 4 h**
	The codon-optimized sequence including *Bsa*I restriction sites was purchased from Eurofins MWG GmbH (www.eurofins.de/de-de.aspx) [1]
**Protein sequence**	MAADTAPADSLYSRMGGEAAVEKAVDVFYERIVADPQLAPFFANVDMKKQRRKQVAFMTYV<br>FGGSGAYEGRDLGASHRRLIREQGMNHHHFDLVAAHLDSTLQELGVAQELKAEAMAIVASA<br>RPLIFGTGEAGAANSAWSHPQFEK
**Protein**	**THB11[a]** (*Cre16.g662750.t1.2*)[a] **37°C, OD_{600} 0.5, 5 h**
**Primer**	for: 5’-ATGGTAGGTCTCAAATGGGCAACTCATGTACTCACCCCC-3’<br>rev: 5’-ATGGTAGGTCTCAGCGCTCACAGCCGCCGAGTCCTCTG-3’
**Protein sequence**	MGNSCTHPLDAVAHLVTEAEIQEAVRSIEEWQKAQAQAFKTGTSTATNAGPLLQRVGGLDV<br>VKKVVELFYRKLYADPQLIKYLHDQDPMHLRAKQSMFVSWLFGPPNVPYTGKSVRIAHLRIIK<br>QRGFSPEDFDLGMKYFEEAMTELGAPEVLRGEVMRRMLPYKDAIFTPAAGDAAEEARWAA<br>EAAAEAARAESPHNGSHAASAVGSKTPSSALHAASANFNGLAAAPDAAAAAAAPVAVAGGS<br>RPGSRQCPFTGGRLSRPASAAVAPTAAAAVAAAAQAAAGSDVSAAVSAGLYPPVPSADNAP<br>VAVAAPVAAAAKPPSRVPSAAAPPDHSVSAMEAELAALGQATPAGAAAAPSVLAAMVEEGA<br>GAVVGEEESDLLGAELAALVAAEDSAAVSAWSHPQFEK
**Protein**	**THB11-N** (*Cre16.g662750.t1.2*) **37°C, OD_{600} 1.2, 3 h**
**Primer**	for: 5’-<br>ATGGTAGGTCTCAGCGCCTCTGGTGGTGGTGAAAACTTGTATTTCCAGGGCAGCGCTG<br>GCACGTCAACTGCGACG-3’<br>rev: 5’-ATGGTAGGTCTCATATCACACAGCCGCCGAGTCCTCTG-3’
**Protein**	MASWSHPQFEKGASGGG*ENLYFQ*↓*G*SA-

**sequence**	**G**TSTATNAGPLLQRVGGLDVVKKVVELFYRKLYADPQLIKYLHDQDPMHLRAKQSMFVSWLFGPPNVPYTGKSVRIAHLRIIKQRGFSPEDFDLGMKYFEEAMTELGAPEVLRGEVMRRMLPYKDAIFTPAAGDAAEEARWAAEAAAEAARAESPHNGSHAASAVGSKTPSSALHAASANFNGLAAAPDAAAAAAAPVAVAGGSRPGSRQCPFTGGRLSRPASAAVAPTAAAAVAAAAQAAAGSDVSAAVSAGLYPPVPSADNAPVAVAAPVAAAAKPPSRVPSAAAPPDHSVSAMEAELAALGQATPAGAAAAPSVLAAMVEEGAGAVVGEEESDLLGAELAALVAAEDSAAV
**Protein**	**THB11-C**[a] (*Cre16.g662750.t1.2*) **37°C, OD_{600} 0.5, 4.5 h**
**Primer**	for: 5’-ATGGTAGGTCTCAAATGGGCAACTCATGTACTCACCCCC-3’<br>rev: 5’-ATGGTAGGTCTCAGCGCTGGCCTCCTCTGCGGCGTCTC-3’
**Protein sequence**	MGNSCTHPLDAVAHLVTEAEIQEAVRSIEEWQKAQAQAFKTGTSTATNAGPLLQRVGGLDVVKKVVELFYRKLYADPQLIKYLHDQDPMHLRAKQSMFVSWLFGPPNVPYTGKSVRIAHLRIIKQRGFSPEDFDLGMKYFEEAMTELGAPEVLRGEVMRRMLPYKDAIFTPAAGDAAEEASAWSHPQFEK
**Protein**	**THB11-NC** (*Cre16.g662750.t1.2*) **25°C, OD_{600} 0.6, 16 h**
**Primer**	for: 5’-ATGGTAGGTCTCAGCGCCTCTGGTGGTGGTGAAAACTTGTATTTCCAGGGCAGCGCTGGCACGTCAACTGCGACG<br>rev: 5’-ATGGTAGGTCTCATATCAGGCCTCCTCTGCGGCGTCTC-3’
**Protein sequence**	MASWSHPQFEKGASGGG*ENLYFQ*↓*gsa*-**g**tstatnaGPLLQRVGGLDVVKKVVELFYRKLYADPQLIKYLHDQDPMHLRAKQSMFVSWLFGPPNVPYTGKSVRIAHLRIIKQRGFSPEDFDLGMKYFEEAMTELGAPEVLRGEVMRRMLPYKDAIFTPAAGDaaeea

[a]These constructs have been reported before [1].

Figure S1

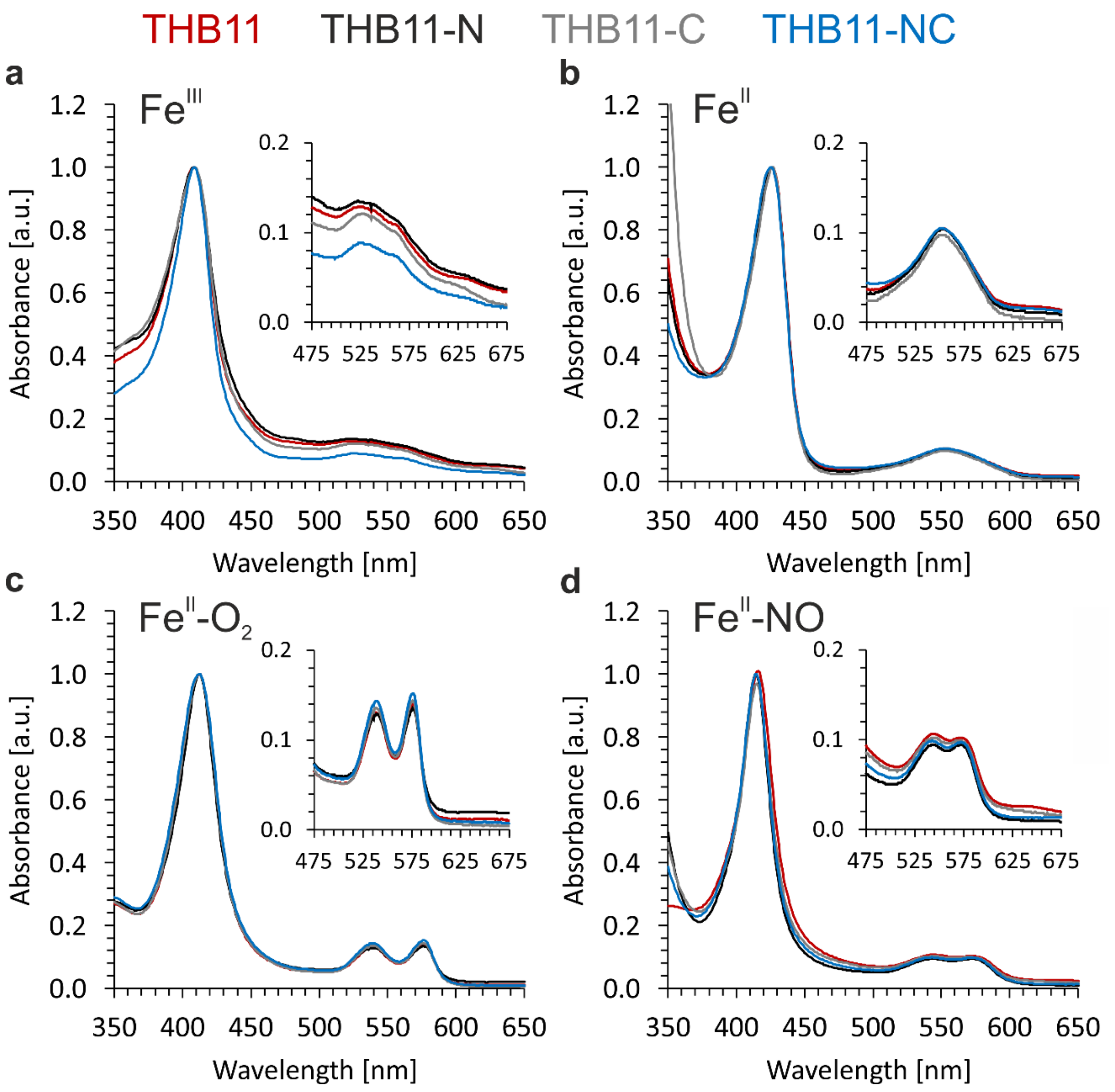

Fig. S1 UV-Vis absorption spectra of full-length recombinant THB11 and its truncated variants are very similar. In all cases, proteins were present in buffer W (100 mM Tris-HCl pH 8.0, 150 mM NaCl, 1 mM EDTA) at a concentration of 10 µM heme. The heme groups were (**a**) oxidized by the addition of 50 µM ferricyanide, (**b**) reduced by 200 µM sodium dithionite (NaDt), (**c**) aerated after reduction and removal of excess NaDt, and (**d**) reduced and treated with 200 µM of diethylamine NONOate diethylammonium salt (DEA NONOate) after the removal of excess NaDt. THB11: full-length protein (red line), THB11-N: N-terminally truncated THB11 (black line); THB11-C: C-terminally truncated THB11 (gray line), THB11-NC: heme-binding domain only (blue line). The spectra were normalized to the Soret peak maxima (set to 1). The insets show enlarged spectra of the region between 475 and 675 nm.

Figure S2

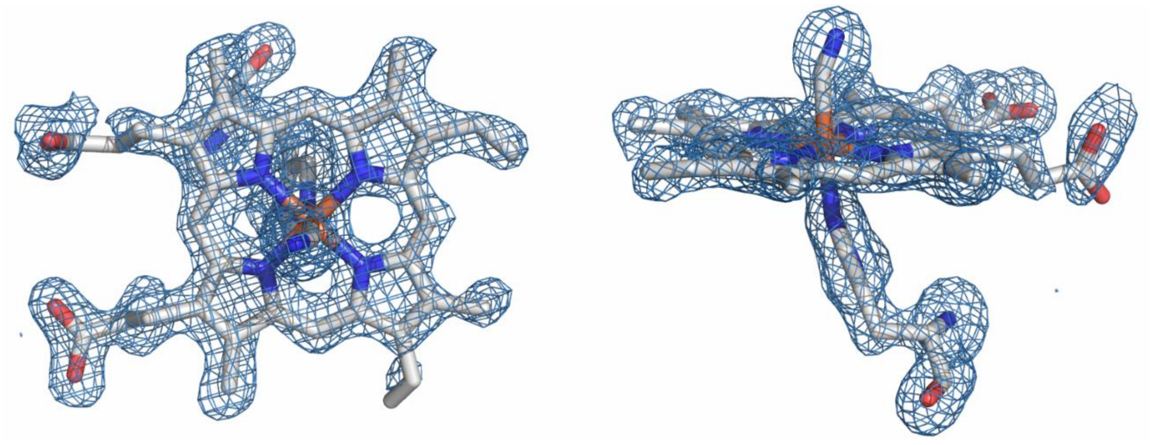

Fig. S2 Simulated annealing omit map of the active site of THB11-NC. The electron density map was calculated omitting the heme group, the CN^{-} ligand and HisF8(81) in the input model. The map is shown in blue mesh contoured at 2σ around the stick models of the omitted groups. Left: View from the distal site, right: view from the side.

Figure S3

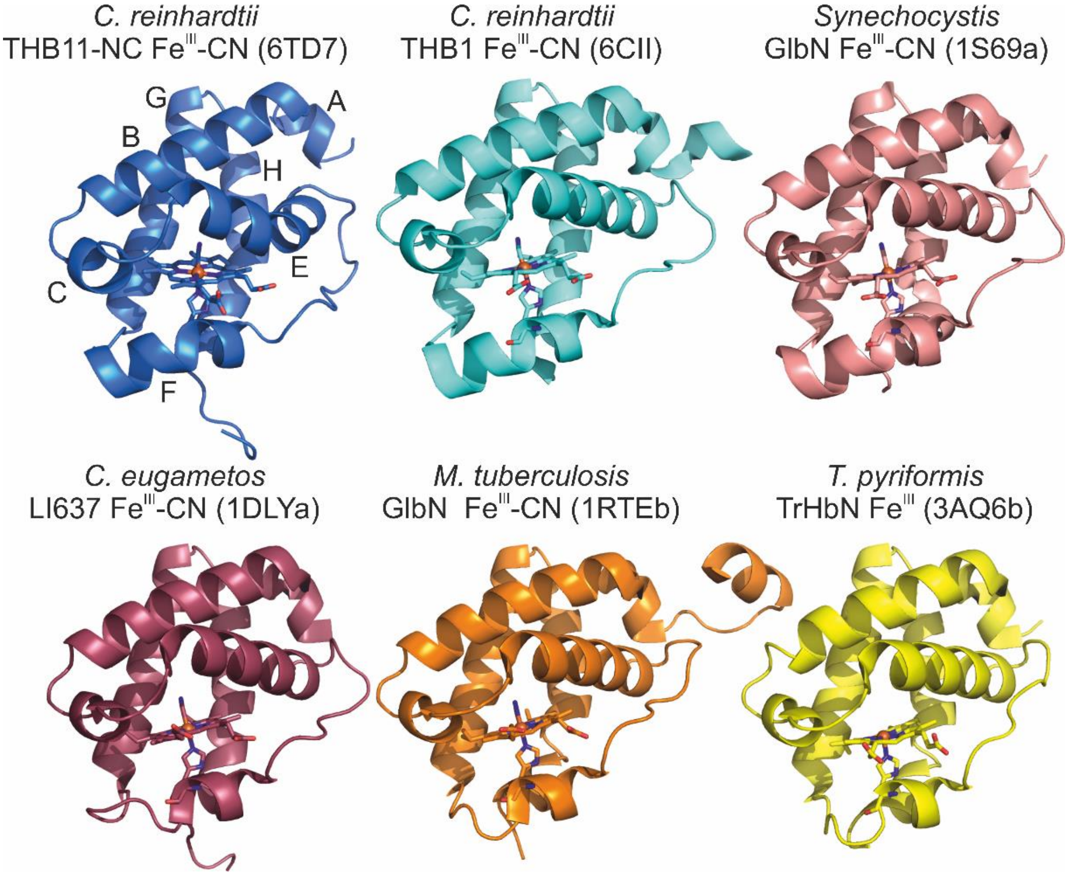

Fig. S3 A comparison of the structures of class I 2/2Hbs shows the conserved overall fold. A kink in helix E is observed in the THB11 heme-binding domain only, whereas the number of F helix turns is variable among the proteins. All structures were aligned to that of THB11-NC in PyMOL. The state of the heme groups and PDB accession numbers are indicated above each cartoon.

Figure S4

```
                     15    2  5    10           1       7   11  15
                AAAAAA  BBBBBBBBBBBBBBBBB   CCCCC   EEEEEEEEEEEEEEEEE
Syn GlbN      MSTLYEKLGGTTAVDLAVDKFYERVLQDDRIKHFFADVDMAKQRAHQKAFLTYAFGGTD-   59
Ce LI637      PSSLFAKLGGREAVEAAVDKFYNKIVADPTVSTYFSNTDMKVQRSKQFAFLAYALGGAS-   59
Mt GlbN       PISIYDKIGGHEAIEVVVEDFYVRVLADDQLSAFFSGTNMSRLKGKQVEFFAAALGGPE-   59
Tp TrHb       PQTIYEKLGGENAMKAAVPLFYKKVLADERVKHFFKNTDMDHQTKQQTDFLTMLLGGPN-   59
THB1-Cr       ADSLYSRMGGEAAVEKAVDVFYERIVADPQLAPFFANVDMKKQRRKQVAFMTYVFGGSG-   59
THB4-Cr       RSTLHAKLGGAAAVAATVDVFYKKLMNDPDLEPFFRGVDMVTLIAKQNRFLAYAFGATT-   59
THB8-Cr       MITLLKRIGGEKGMKDVVEMFYRKIYANERLRHFLKDKDVSLLRSKQAAFMTWLFGPSTA   60
THB9-Cr       NGPLLDRMGGADYVKRVAERFYHKLYADEQLAPFLGEHDVVSLRAKQNAFLIWAFGPPNR   60
THB10-Cr      KTPLLARVGGIDNVKRVVEGFYRRLYADERMDKYLSGFDMPYLRAKQSAFMGWLFGPASV   60
THB11-Cr      AGPLLQRVGGLDVVKKVVELFYRKLYADPQLIKYLHDQDPMHLRAKQSMFVSWLFGPPNV   60
THB12-Cr      TGPLLQRVGGSDVVKHVVELFYRHLYADPSLIKFLHDQEMTHLRAKQSAFISWLFGPPNV   60
                 :  ::**   :  ..  ** ::  :  :  ::   :      :*  *.   :*

                        8             5   9  12  16         5  8   12
                   FFFFFFF        GGGGGGGGGGGGGGGGGGG  HHHHHHHHHHHHHHHHHHH
Syn GlbN      KYDGRYMREAHKELVENHGLNGEHFDAVAEDLLATLKEMGVPEDLIAEVAAVAGAPAHKR   119
Ce LI637      EWKGKDMRTAHKDLVPH--LSDVHFQAVARHLSDTLTELGVPPEDITDAMAVVASTRTEV   117
Mt GlbN       PYTGAPMKQVHQ----GRGITMHHFSLVAGHLADALTAAGVPSETITEILGVIAPLAVDV   115
Tp TrHb       HYKGKNMTEAHKGMN----LQNLHFDAIIENLAATLKELGVTDAVINEAAKVIEHTRKDM   115
THB1-Cr       AYEGRDLGASHRRLIREQGMNHHHFDLVAAHLDSTLQELGVAQELKAEAMAIVASARP--   117
THB4-Cr       HYHGKDIVMGHAHLIINRGLNLTHFDKVAGHFVDSLKEMGVGQELIDEAAGVLIGVRPLF   119
THB8-Cr       TYTGRSVRCAHLRMIKQRGFSEDDFLLGMSCFEEAMRDYGAPQRLTNEIMAKILPFKDII   120
THB9-Cr       PYPGRHLRTAHLRLIKQKGFTVEHFEMGLKHFEEALKEMDAPPSVVRDALMKLRPYKTVV   120
THB10-Cr      PYSGKHLRTVHLKMIKQQGFSPEDFEVGMDYFEESMKELGAPETIISEVMSKIRPFKEVI   120
THB11-Cr      PYTGKSVRIAHLRIIKQRGFSPEDFDLGMKYFEEAMTELGAPEVLRGEVMRRMLPYKDAI   120
THB12-Cr      PYTGKSVRIAHLRIIKQRGFSPEDFDLGMKYFAESMRELGAPEILINEVLRRITPYKDAI   120
               : *  :   *        :   .*      :  ::   ..      :

              H
Syn GlbN      DVLNQ      124
Ce LI637      LNM--      120
Mt GlbN       TSG--      118
Tp TrHb       LGK--      118
THB1-Cr       LIFGT      122
THB4-Cr       DPE--      122
THB8-Cr       FSPS-      124
THB9-Cr       FTPS-      124
THB10-Cr      FTPN-      124
THB11-Cr      FTPA-      124
THB12-Cr      FTPS-      124
```

Fig. S4 Sequence alignment of class I 2/2Hbs. The heme-binding domains of *Chlamydomonas* (Cr) THB8 to THB11, which form a phylogenetic cluster [2,3], were aligned to selected class I 2/2Hbs, for which structural information is available: *Synechocystis* sp. PCC 6803 GlbN (Syn GlbN; UniProt ID P73925), *C. eugametos/moewusii* LI637 (Ce LI637; UniProt ID Q08753), *Mycobacterium tuberculosis* GlbN (Mt GlbN; NCBI WP_075845202), *Tetrahymena pyriformis* TrHbN (Tp TrHb; UniProt ID P17724), as well as THB1 and THB4 from *Chlamydomonas*. Alignments were done in Clustal Omega, employing default settings, but keeping the sequence input order. Helices are indicated above the alignment, employing the Perutz nomenclature. Functionally important residues that are discussed in the main text are colored and written in bold letters and indicated by their Perutz numbers above the helix letters. **Black/white letters:** heme-coordinating HisF8; **turquoise** (B10, E7, E11): ligand stabilization through H-bond networks; **dark red/white letters** (E10): employed for hexacoordination in Syn GlbN [4,5], THB1-Cr and THB4-Cr [6,7]; **green:** important tunnel residues in *M. tuberculosis* GlbN (A15, B2, B5, G16: long tunnel (LT)

aperture; G5, G9, H8, H11, H12: short tunnel (ST) opening; E15, G12: ST-LT intersection and E15 gate) [8,9]; **gray:** Gly motives; **yellow:** residues having alternative conformations in the THB11-NC crystal structure. Gene IDs of the *Chlamydomonas* 2/2Hbs are Cre14.g615400 (THB1), Cre04.g218750 (THB4), Cre16.g661200 (THB8), Cre16.g661250 (THB9), Cre16.g661300 (THB10), Cre16.g662750 (THB11) and Cre16.g663000 (THB12).

Figure S5

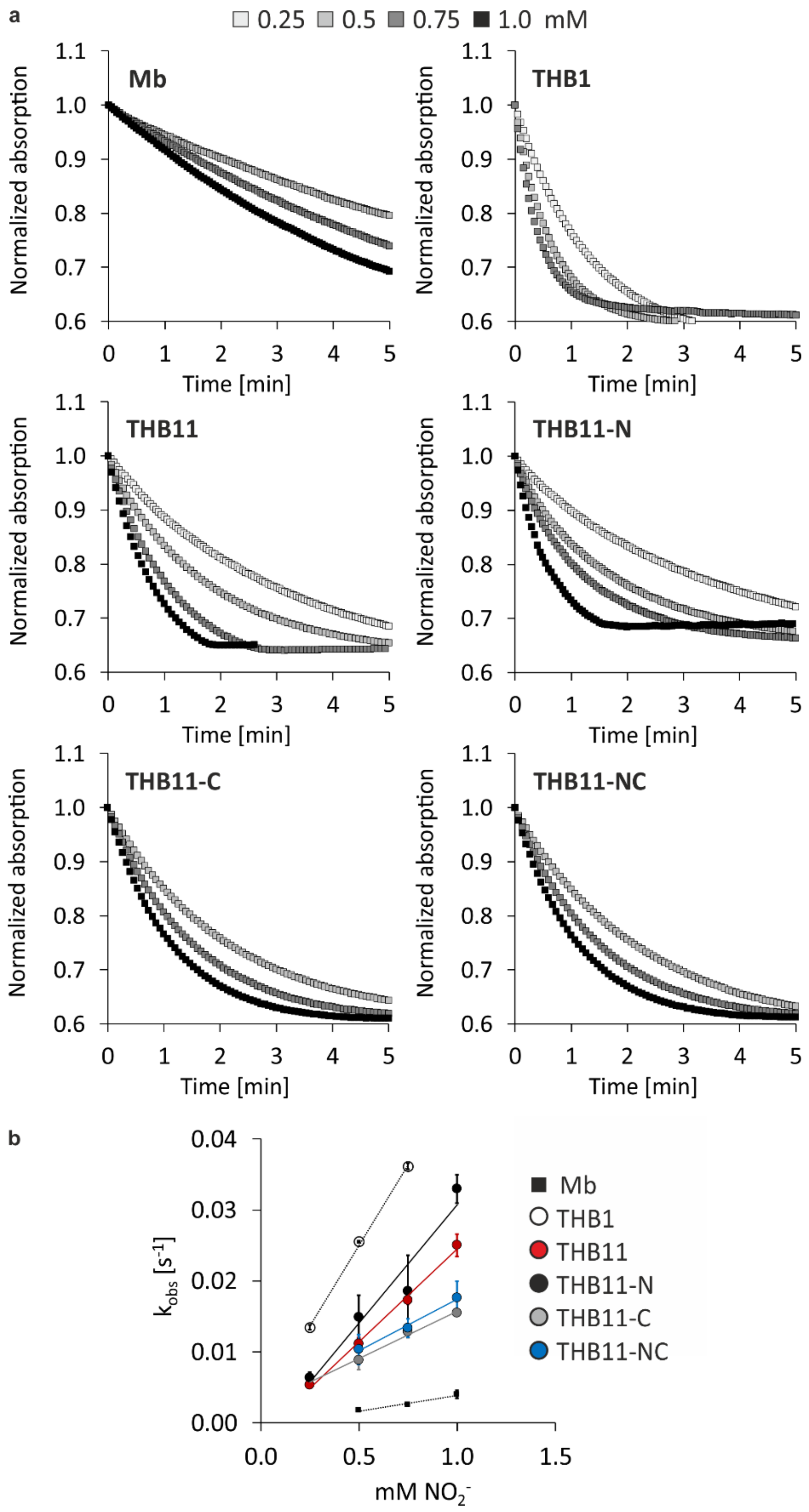

Fig. S5 Kinetics of the reactions of THB11 variants with nitrite. The recombinant *Chlamydomonas* THB11 length variants as well as horse heart myoglobin (Mb) and

Chlamydomonas THB1 were incubated in anoxic 50 mM HEPES buffer, pH 7.4, 100 mM NaCl, supplemented with 600 µM sodium dithionite at concentrations of 5 µM heme in their deoxy (heme-Fe^{II}) states. Reactions were started by adding anoxic potassium nitrite to the indicated final concentrations. Note that the slower proteins (Mb, THB11-C, THB11-NC) were not tested with 0.25 mM nitrite, whereas the fast THB1 was not analyzed with 1 mM nitrite. Experiments were done in technical triplicates per nitrite concentration from at least two independent protein batches. **a:** The decreases of the Soret peak maxima of the deoxy globins were followed spectroscopically at 20°C after the addition of the indicated concentrations of anoxic potassium nitrite. Representative traces of individual experiments are shown in each case. **b:** Exponential decay fits of the absorption changes in time yielded the pseudo first order rate constants k_{obs}. The data points indicate the means of k_{obs} values per nitrite concentration, which were in turn obtained by averaging the k_{obs} values per technical triplicate. Lines represent linear fits, error bars indicate the standard deviation.

Figure S6

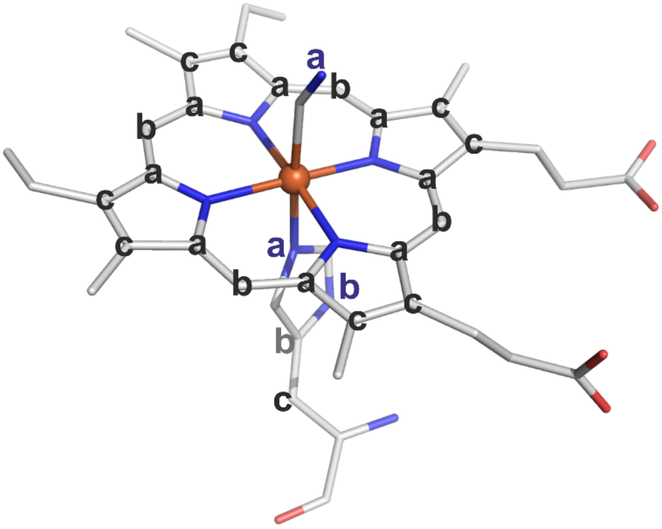

	THB11 EXAFS	THB11-C EXAFS	THB11-NC crystal
		Å	
Fe..N_{heme}	2.00	2.02	~2.05
Fe..N_{HisF8}	2.48	2.35	2.2
Fe..O_2/CN	1.90	1.91	2.2
Fe..a	3.02	3.04	~3.08 (~2.9)
Fe..b	3.36	3.35	~3.45 (~3.55)
Fe..c	4.27	4.28	~4.28 (~4.43)

Fig. S6 Comparison of interatomic distances measured in the THB11-NC crystal structure with those obtained by EXAFS analyses from THB11 and THB11-C. To the left, atoms are labeled within the heme group of THB11-NC according to the first (a), second (b) and third (c) sphere. The table to the right summarizes the distances in Å obtained from EXAFS simulations from full-length THB11 and THB11-C in their oxy-forms [1] and those measured in the cyanomet THB11-NC crystal structure in this study. Fe..N_{heme}, Fe..N_{HisF8} and Fe..O_2/CN indicate the direct distances of the heme-Fe to the pyrrole N atoms, the coordinating HisF8 Nε atom and the ligand (O_2 in EXAFS analyses, CN^- in this study), respectively. Except Fe..N_{HisF8} and Fe..O_2/CN, distances determined for THB11-NC in PyMOL were averaged, indicated by a tilde sign. Because the Fe..N_{HisF8} and Fe..O_2/CN distances showed larger deviations, the Fe..a, Fe..b and Fe..c distances were calculated both excluding and including the axial ligand distances, and the latter are indicated in brackets.

Figure S7

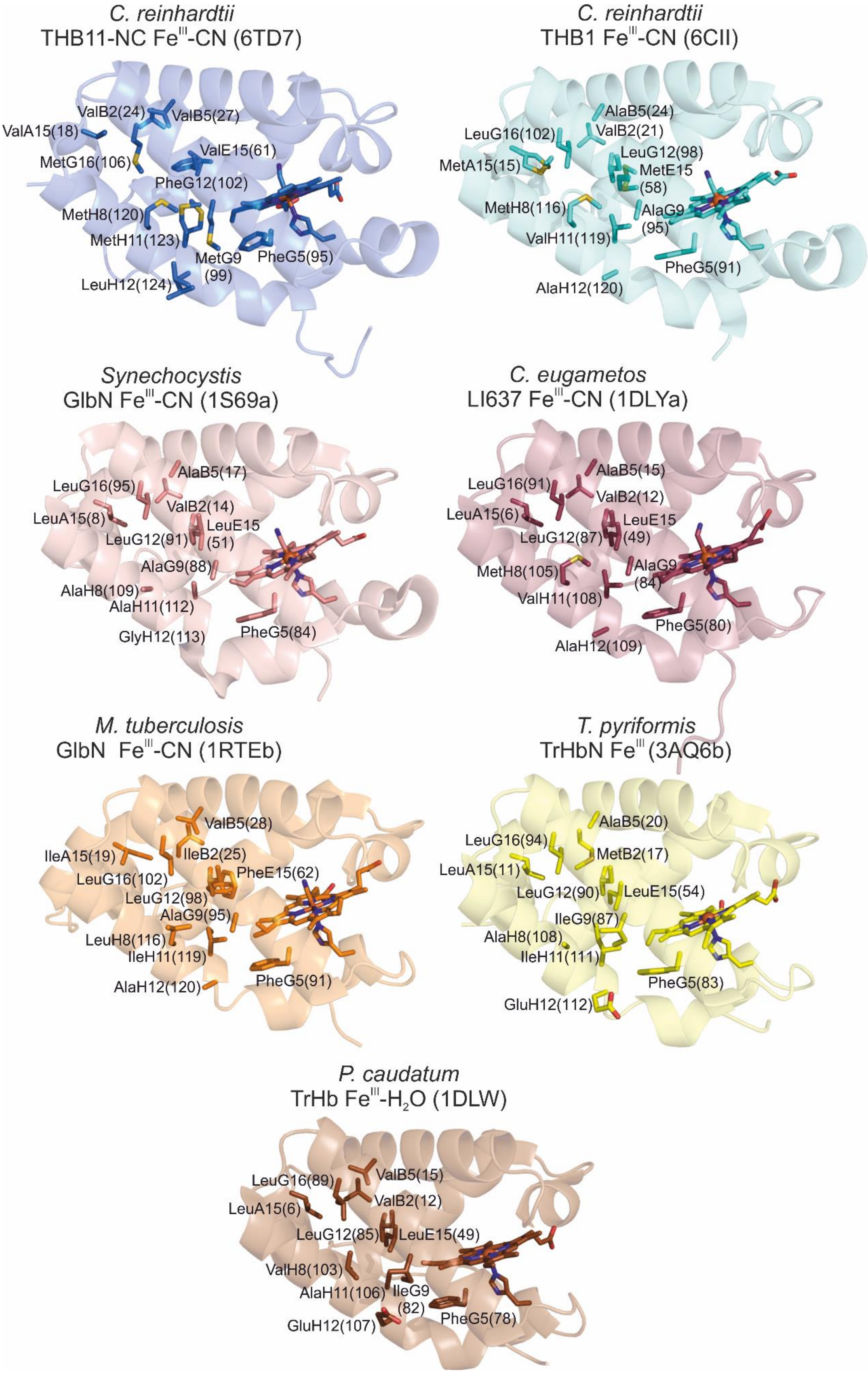
C. reinhardtii
THB11-NC Fe^{III}-CN (6TD7)
ValB2(24)
ValB5(27)
ValA15(18)
ValE15(61)
MetG16(106)
PheG12(102)
MetH8(120)
MetH11(123)
MetG9
(99)
PheG5(95)
LeuH12(124)
C. reinhardtii
THB1 Fe^{III}-CN (6CII)
AlaB5(24)
LeuG16(102)
ValB2(21)
LeuG12(98)
MetA15(15)
MetE15
(58)
MetH8(116)
AlaG9
(95)
ValH11(119)
PheG5(91)
AlaH12(120)
Synechocystis
GlbN Fe^{III}-CN (1S69a)
AlaB5(17)
LeuG16(95)
ValB2(14)
LeuA15(8)
LeuE15
(51)
LeuG12(91)
AlaG9(88)
AlaH8(109)
AlaH11(112)
GlyH12(113)
PheG5(84)
C. eugametos
LI637 Fe^{III}-CN (1DLYa)
AlaB5(15)
LeuG16(91)
ValB2(12)
LeuA15(6)
LeuE15
LeuG12(87)
(49)
MetH8(105)
AlaG9
(84)
ValH11(108)
PheG5(80)
AlaH12(109)
M. tuberculosis
GlbN Fe^{III}-CN (1RTEb)
ValB5(28)
IleA15(19)
IleB2(25)
LeuG16(102)
PheE15(62)
LeuG12(98)
AlaG9(95)
LeuH8(116)
IleH11(119)
AlaH12(120)
PheG5(91)
T. pyriformis
TrHbN Fe^{III} (3AQ6b)
AlaB5(20)
LeuG16(94)
MetB2(17)
LeuA15(11)
LeuG12(90)
LeuE15(54)
IleG9(87)
AlaH8(108)
IleH11(111)
PheG5(83)
GluH12(112)
P. caudatum
TrHb Fe^{III}-H_2O (1DLW)
LeuG16(89)
ValB5(15)
ValB2(12)
LeuA15(6)
LeuG12(85)
LeuE15(49)
ValH8(103)
AlaH11(106)
IleG9
(82)
PheG5(78)
GluH12(107)

Fig. S7 Residues reported to line the long (LT) and short tunnels (ST) in class I 2/2Hbs. The residues are represented as sticks and labeled according to the Perutz nomenclature. Their positions in the sequences associated with the respective structures are indicated in brackets. The states of the heme-Fe and the PDB IDs are shown above each Hb representation.

Figure S8

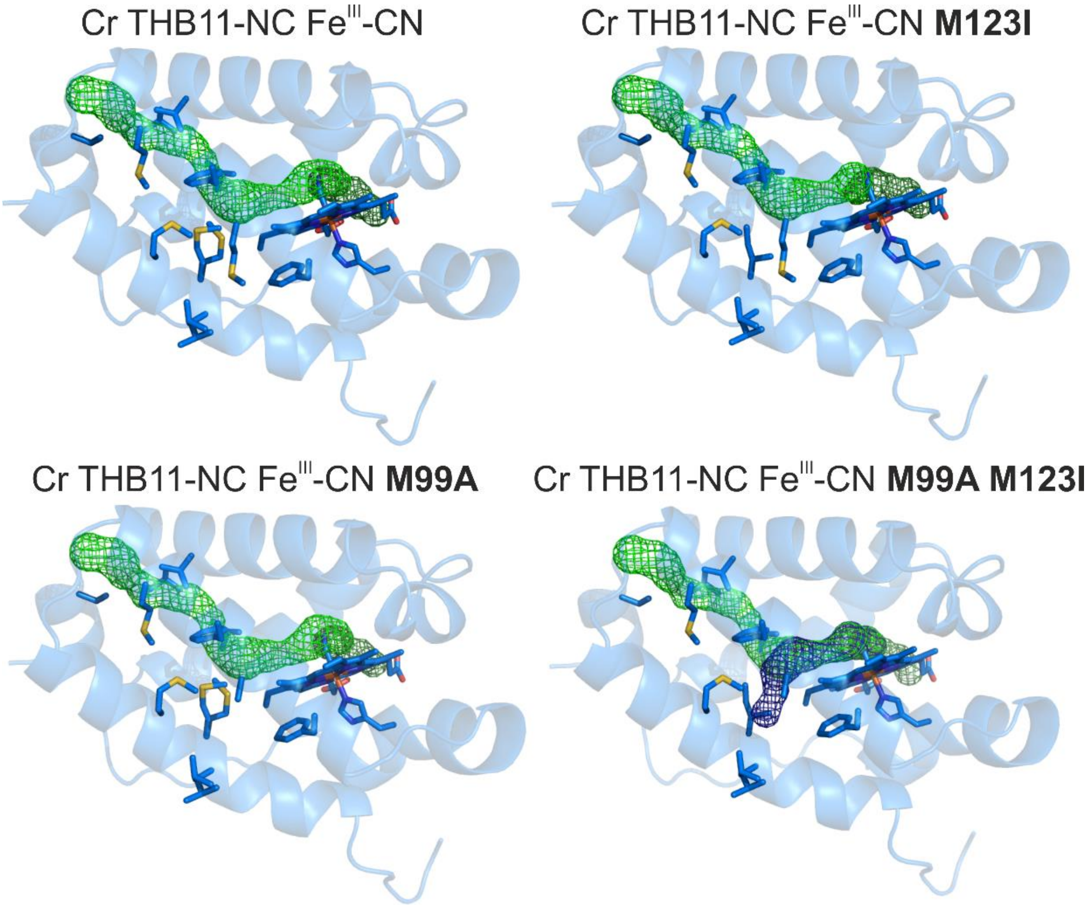

Fig. S8 A short tunnel can be computed in *in silico* mutagenized THB11-NC. Residues Met99, Met123 or both were exchanged in PyMOL by the residues found at the topological positions in *M. tuberculosis* GlbN. Caver was employed to compute tunnels, applying default settings and excluding the CN^- ligand.

References cited in the supplementary material

1. Huwald D, Schrapers P, Kositzki R, Haumann M, Hemschemeier A (2015) Characterization of unusual truncated hemoglobins of *Chlamydomonas reinhardtii* suggests specialized functions. Planta 242:167-185
2. Hemschemeier A, Düner M, Casero D, Merchant SS, Winkler M, Happe T (2013) Hypoxic survival requires a 2-on-2 hemoglobin in a process involving nitric oxide. Proc Natl Acad Sci U S A 110:10854-10859
3. Johnson EA, Lecomte JTJ (2015) The Haemoglobins of Algae. In: Robert KP (ed) Advances in Microbial Physiology, vol Volume 67. Academic Press, pp 177-234
4. Scott NL, Lecomte JT (2000) Cloning, expression, purification, and preliminary characterization of a putative hemoglobin from the cyanobacterium *Synechocystis* sp. PCC 6803. Protein Sci 9:587-597
5. Hvitved AN, Trent JT, 3rd, Premer SA, Hargrove MS (2001) Ligand binding and hexacoordination in *Synechocystis* hemoglobin. J Biol Chem 276:34714-34721
6. Rice SL, Boucher LE, Schlessman JL, Preimesberger MR, Bosch J, Lecomte JT (2015) Structure of *Chlamydomonas reinhardtii* THB1, a group 1 truncated hemoglobin with a rare histidine-lysine heme ligation. Acta Crystallogr F Struct Biol Commun 71:718-725
7. Johnson EA, Russo MM, Nye DB, Schlessman JL, Lecomte JTJ (2018) Lysine as a heme iron ligand: A property common to three truncated hemoglobins from *Chlamydomonas reinhardtii*. Biochim Biophys Acta Gen Subj 1862:2660-2673
8. Milani M, Pesce A, Ouellet Y, Ascenzi P, Guertin M, Bolognesi M (2001) *Mycobacterium tuberculosis* hemoglobin N displays a protein tunnel suited for O_2 diffusion to the heme. EMBO J 20:3902-3909
9. Milani M, Pesce A, Ouellet Y, Dewilde S, Friedman J, Ascenzi P, Guertin M, Bolognesi M (2004) Heme-ligand tunneling in group I truncated hemoglobins. J Biol Chem 279:21520-21525
